# Artificial gravity during prolonged bed rest preserves resting metabolic rate but not muscle function: Evidence from the BRACE study

**DOI:** 10.1113/JP290788

**Published:** 2026-05-24

**Authors:** Saul Martin‐Rodriguez, Mirko Mandić, Claire Laurens, Marie‐Pierre Bareille, Rebecca Billette de Villemeur, Rodrigo Fernandez‐Gonzalo, Chantal Simon

**Affiliations:** ^1^ Department of Laboratory Medicine Division of Clinical Physiology Karolinska Institutet Stockholm Sweden; ^2^ Unit of Clinical Physiology Karolinska University Hospital Stockholm Sweden; ^3^ Metabolic and Cardiovascular Research Institute University of Toulouse, UMR 1297 Toulouse France; ^4^ Institute for Space Medicine and Physiology (MEDES) Toulouse France; ^5^ CarMen Laboratory, INSERM 1060, INRAE 1397 University of Lyon Oullins France

**Keywords:** artificial gravity, bed rest, muscle function, resting metabolic rate, substrate utilization

## Abstract

**Abstract:**

Prolonged head‐down tilt bed rest induces metabolic and neuromuscular deconditioning. This study examined whether supine cycling exercise, with/without artificial gravity (AG), preserves resting metabolic rate (RMR) during 60 days of bed rest, and whether RMR responses align with integrated multivariate physiological adaptations. Twenty‐four healthy men were randomly assigned to control (C), cycling (EX) or cycling plus AG (EX‐AG). RMR, substrate oxidation, body composition (dual‐energy X‐ray absorptiometry/magnetic resonance imaging), muscle function and maximal cycling power were assessed before and after bed rest. Linear mixed models evaluated group‐by‐time effects. Principal component analysis (PCA) and sparse partial least squares (sPLS) regression examined multivariate correlates of ΔRMR. RMR responses differed between groups (interaction *P* = 0.029), with EX‐AG preventing the decline observed in C (EX‐AG *vs*. C ΔΔ = +133 kcal day^−1^, *P* = 0.017). Fat oxidation remained stable in EX‐AG but declined in C and EX. Both interventions attenuated lower‐body fat‐free mass loss, with greater preservation under EX‐AG, yet neuromuscular function deteriorated similarly across groups. Maximal cycling power exhibited a significant interaction (*P* < 0.001), declining in C, being maintained in EX and increasing in EX‐AG. PCA revealed two dominant axes: PC1 (25.9% variance) reflected preservation of fat‐free tissues, whereas PC2 (17.9%) captured gains in abdominal adiposity. EX‐AG participants clustered toward higher PC1 values, indicating tissue preservation. The sPLS model (*r* = 0.72) identified respiratory ratio changes (ΔRQ) as the dominant contributor. Exercise combined with AG preserves resting metabolism but does not prevent neuromuscular decline. Multivariate analyses indicate that substrate utilization and preservation of metabolically active lean compartments underpin RMR maintenance during unloading.

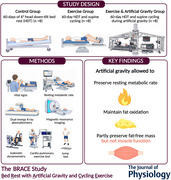

**Key points:**

- Cycling exercise combined with artificial gravity appeared to preserve resting metabolic rate during long‐term (60 days) head‐down tilt bed rest.
- Cycling with/without artificial gravity failed to prevent declines in maximal voluntary contraction or jump performance induced by 60 days of bed rest, although it preserved cycling power.
- We show that metabolic and neuromuscular adaptations to bed rest may be partially dissociated, with artificial gravity primarily sustaining basal metabolic regulation while failing to maintain skeletal muscle contractile function.
- The dissociation between metabolic and mechanical adaptations reported here provides valuable insight into the regulation of energy expenditure and muscle function in other contexts of severe inactivity, such as immobilization or ageing.

## Graphical abstract

**Abstract figure legend** This study examined whether supine cycling exercise, with or without artificial gravity, preserves resting metabolic rate (RMR) during 60 days of head‐down tilt bed rest, and whether RMR responses align with integrated multivariate physiological adaptations. Twenty‐four healthy men were randomly assigned to a control group, a supine cycling group or a supine cycling during artificial gravity group. RMR, substrate oxidation, body composition (dual‐energy X‐ray absorptiometry/magnetic resonance imaging), muscle function and maximal cycling power were assessed before and after bed rest. Exercise during artificial gravity preserved RMR and maintained fat oxidation but did not prevent neuromuscular decline. Multivariate analyses indicate that substrate utilization and preservation of metabolically active lean compartments underpin RMR maintenance during bed rest.

## Introduction

Prolonged 6° head‐down tilt bed rest is a well‐established terrestrial model to study the physiological effects of microgravity (Hargens & Vico, 2016). It induces a cascade of adaptations involving muscle atrophy, loss of strength and reductions in resting metabolic rate (RMR), reflecting the overall decline in the metabolic demand associated with disuse (Eggelbusch et al., 2024; Laurens et al., 2019). The skeletal muscle system, which accounts for approximately 20–25% of total daily energy expenditure (Zurlo et al., 1990), is particularly vulnerable to bed rest, showing marked decrements in muscle mass and function. Losses in fat‐free mass (FFM) explain part of the reduction in RMR (Sparti et al., 1997). However, reductions in metabolic rate during bed rest have been reported to exceed that expected from FFM loss alone, suggesting additional metabolic suppression that persists after adjustment for changes in body composition (Bergouignan et al., 2006; Bourdier et al., 2025) and may reflect alterations in tissue metabolic activity or neuroendocrine regulation.

This adaptative energy‐conservation response, also observed in caloric restriction and hypokinetic (low‐activity) states, complicates the maintenance of energy balance during and after periods of inactivity. Importantly, regulation of RMR during bed rest is influenced not only by quantitative changes in FFM but also by qualitative alterations in skeletal muscle and in other tissues, including muscle fat infiltration, substrate utilization and hormonal regulation (Eggelbusch et al., 2024).

Given the pronounced physiological deconditioning induced by prolonged bed rest, a wide range of countermeasures (i.e. structured interventions implemented to limit physiological deconditioning during bed rest) have been developed to attenuate muscle atrophy, declines in cardiorespiratory fitness and metabolic dysregulation (Fernandez‐Gonzalo et al., 2026; Scott et al., 2023). For example, in a 21 day bed rest study, a resistive‐vibration exercise protocol attenuated the reduction in RMR by approximately 40%, while lean mass preservation remained incomplete (Bourdier et al., 2025).

Although exercise remains the most well‐established countermeasure, it does not fully prevent the metabolic and musculoskeletal deconditioning induced by unloading. Artificial gravity (AG), generated by short‐arm centrifugation, has therefore been proposed as a promising adjunct to exercise, aiming to provide both mechanical loading and cardiovascular stimulation (Clement et al., 2015). Recent findings showed that, despite a moderate preservation of lower‐limb FFM with cycling exercise alone or combined with AG, muscle atrophy and increases in intramuscular fat infiltration still occurred (Mandic et al., 2025). This indicates that cycling exercise and AG only partially protect skeletal muscle morphology under unloading conditions. Despite these and other recent advances (Arbeille et al., 2024; Hedge et al., 2025; Mandic et al., 2025), clarifying whether concurrent AG and exercise can preserve both body composition and metabolic function during prolonged bed rest, and whether these effects mirror muscle structural adaptations, represent key unanswered questions.

The present study aimed to explore the effects of prolonged bed rest, with and without cycling exercise and AG countermeasures, on RMR and its association with changes in body composition, specifically in muscle mass, and muscle function. We hypothesized that RMR would decrease following prolonged bed rest, despite the countermeasure‐induced partial preservation of lean tissue mass, suggesting that metabolic adaptations extend beyond changes in body composition.

## Methods

### Ethical approval

The study was designed and performed according to the Declaration of Helsinki and was approved by the Comité de Protection des Personnes Île‐de‐France VI on 19 December 2022 and authorized by the French National Agency for Medicines and Health Products Safety (ANSM) on 11 January 2023. All participants received verbal and written information about the study and provided written informed consent prior to enrolment. The study was registered in France under the ID‐RCB number 2022‐A02074‐39 and in the ClinicalTrials.gov database under the identifier NCT06544213. In addition, the handling and analysis of data were approved by the Swedish Ethical Review Authority (reference number 2022‐06455‐01).

### Overview of research design

This study is part of the Bed Rest with Artificial Gravity and Cycling Exercise (BRACE) project, jointly supported by the European Space Agency (ESA) and the French Space Agency (CNES). Twenty‐four healthy male participants were enrolled in the study, which took place at the MEDES Space Clinic (Toulouse, France). The experimental protocol comprised three distinct phases: (i) a 14 day baseline data collection period (BDC‐14 to BDC‐1) conducted under ambulatory but confined conditions with structured physical activity; (ii) a 60 day head‐down tilt bed rest (HDT) phase at −6°, performed either with or without countermeasures (HDT1 to HDT60); and (iii) a 14 day post‐bed‐rest recovery period (R+0 to R+13) under ambulatory confinement. A comprehensive description of the study design, recruitment strategy and selection procedures has been published elsewhere (Mandic et al., 2025).

### Participants and facilities

Participants were randomly allocated to one of three experimental groups, each comprising eight volunteers (values are mean ± SD): a control group [C; age 29 ± 7 years, height 172 ± 7 cm, body mass index (BMI) 24 ± 2 kg m^−^^2^], an exercise‐only group (EX; age 30 ± 5 years, height 178 ± 4 cm, BMI 24 ± 2 kg m^−^^2^) and an exercise plus AG group (EX‐AG age 30 ± 6 years, height 177 ± 7 cm, BMI 24 ± 2 kg m^−^^2^). During the HDT period, the C group did not perform any countermeasure protocol; the EX‐group completed a 30 min supine cycling regimen 6 days per week, whereas the EX‐AG group performed the same cycling protocol while simultaneously exposed to AG. During the BDC period, participants followed an individualized and supervised physical activity programme on non‐testing days to maintain fitness before bed rest. Activity targets were based on each participant's RMR. Approximately 40% of the prescribed activity‐related energy expenditure was achieved through structured exercise sessions (treadmill and cycle ergometry with individualized intensity), with the remainder achieved through monitored walking within the facility (Polar Loop activity tracker). All sessions were supervised by MEDES staff. Following the HDT period, participants entered a structured 14 day rehabilitation programme based on ESA post‐flight reconditioning guidelines. The programme consisted of daily supervised sessions (∼45 min) designed to progressively restore postural control, joint stability and muscular function. It followed three sequential phases: (1) retraining anti‐gravity muscles and balance (‘back to gravity’); (2) strength and proprioception training to support safe resumption of daily activities (‘back to function’); and (3) rebuilding power and endurance (‘back to sport’). Although the overall rehabilitation framework was identical for all participants, progression and exercise intensity were individually adjusted according to functional capacity, tolerance and scheduling constraints related to scientific assessments. Detailed descriptive information on the participants has been published elsewhere (Mandic et al., 2025).

### Nutritional prescription

Dietary intake was rigorously controlled throughout the study. All meals were standardized, weighed before and after consumption, and their nutritional composition was calculated using validated French food composition databases (Ciqual; https://ciqual.anses.fr/) supplemented by manufacturer‐provided data when required. Participants received four meals per day (breakfast, lunch, 4 p.m. snack and dinner) based on a 10 day rotating menu. Energy prescriptions were individualized and derived from each participant's RMR measured by indirect calorimetry upon entry into the facility (BDC‐14), and expressed as physical activity level (PAL). Prescriptions were adjusted according to study phase and countermeasure‐related energy expenditure.

During the ambulatory phases (BDC and recovery), total energy intake was set at PAL = 1.60 (160% of RMR) for all groups. Meals were fully prescribed and complete consumption was required; dinner portions were adjusted when necessary to compensate for any uneaten food earlier in the day in order to maintain daily energy targets.

During the initial bed rest phase (HDT1–HDT10), basal energy requirements were set at PAL = 1.30 (130% of RMR). Participants assigned to the exercise countermeasure groups received additional calories corresponding to the estimated energy cost of each training session. Training energy expenditure was individually estimated from each participant's ${\dot{V}}_{O_{2}\mathrm{peak}}$, assessed in the supine position during the BDC phase (Mandic et al., 2025), and applied identically in both exercise groups. Meals remained fully prescribed and complete consumption was required.

From HDT11 to HDT49, controlled flexibility in energy intake (90–110% of prescribed target) was introduced in accordance with the protocol of one scientific team. If energy intake fell below 90% of the prescribed target for two consecutive days, participants were required to consume 100% of their prescribed energy requirement on the third day to maintain compliance with the energy needs. As monitoring of body mass (BM) and composition during the first 10 days of HDT indicated that basal energy needs had been underestimated, the basal PAL during this phase was revised from 1.30 to 1.42 (142% of RMR). Exercise‐related caloric compensation continued to be applied in the EX and EX‐AG groups.

During the late bed‐rest testing phase (HDT50–HDT60), flexibility was discontinued, to ensure strict metabolic standardization. A fixed prescription was reinstate using a basal PAL of 1.49 based on cumulative energy balance analyses. Complete consumption was again required, with dinner adjustments implemented when necessary.

Macronutrient composition was standardized across groups throughout the study. Protein intake was prescribed at 1.0–1.2 g kg^−^^1^ day^−1^ of baseline BM and increased to 1.3 g kg^−^^1^ day^−1^ during HDT11–HDT49 to ensure adequate intake even at 90% energy coverage. Total fat contributed approximately 35% of total energy, and carbohydrate intake accounted for the remaining energy (approximately 49–51% across phases). Although total caloric intake differed between groups due to differences in baseline RMR and exercise‐related compensation, macronutrient distribution remained highly comparable between groups across all study phases (see Results). All meals complied with the ESA micronutrient recommendations.

### Baseline data collection

During the BDC phase, participants completed two cardiopulmonary exercise tests: one in the upright position and one in the supine position. Both assessments followed an identical incremental cycling protocol in which participants pedaled at 50 W for 3 mins at a cadence of at least 70 revolutions per min, after which the workload increased by 25 W every minute until volitional exhaustion. Data obtained from the supine test were subsequently used to design individualized training programmes for the two countermeasure groups (EX and EX‐AG). Specifically, a linear regression between workload (in W) and oxygen uptake (${\dot{V}}_{O_{2}}$) was calculated over the range from 75 W to the last completed workload (i.e. peak load – 25 W) to determine the target cycling load corresponding to a defined percentage of each participant's ${\dot{V}}_{O_{2}\mathrm{peak}}$.

Orthostatic tolerance (OT) and presyncope (PS) assessments were conducted using the ESA's Short‐Arm Human Centrifuge (SAHC; MEDES, Toulouse, France). The centrifugation protocol began at 0.6 Gz (measured at the body's centre of gravity) and was maintained for 10 mins, after which the load was increased in increments of 0.1 Gz every 3 mins until PS occurred. PS was identified based on cardiovascular variables (blood pressure and heart rate) and participant‐reported symptoms. OT was defined as the Gz level at which physiological signs of central hypovolaemia first appeared, characterized by a 20% increase in heart rate from baseline (determined via quadratic fitting of the heart‐rate response curve) and by evidence of peripheral vasoconstriction, indicated by a marked and consistent decrease in oxygenated haemoglobin in the calf region (Ap et al., 2025).

### Countermeasures

Exercise countermeasures were implemented using a supine cycle ergometer (Angio CPET, Lode, Groningen, The Netherlands) and were identical in overall structure for the EX and EX‐AG groups. Participants’ feet were securely strapped to the pedals to ensure consistent force transmission during supine cycling. Exercise intensity was individually prescribed based on ${\dot{V}}_{O_{2}\mathrm{peak}}$ values obtained from the supine incremental cycling test conducted during BDC. Each exercise session followed a standardized interval‐based protocol. After a 5 min warm‐up performed at 40% of individual ${\dot{V}}_{O_{2}\mathrm{peak}}$, participants completed six 2 min work intervals at intensities ranging from 65% to 80% of ${\dot{V}}_{O_{2}\mathrm{peak}}$. Each high‐intensity interval was separated by a 2 min period of active recovery at 40% of ${\dot{V}}_{O_{2}\mathrm{peak}}$. Sessions concluded with a 3 min cool‐down at the same intensity.

In the EX‐AG group, cycling exercise was performed during exposure to AG generated by a short‐arm human centrifuge. The AG profile was individually tailored based on OT and PS thresholds determined during pre‐bed‐rest testing, as previously described within the BRACE study framework (Mandic et al., 2025). For each participant, a personalized Gz profile was constructed, beginning at a level 0.15 Gz below OT and progressively increasing in synchrony with the cycling protocol to a maximum corresponding to 70% of the individual tolerance range. Gravitational load was subsequently reduced in a stepwise manner until completion of the session. The direction of centrifuge rotation was alternated between sessions to minimize directional bias. The centrifugation protocol, based on the individual OT and PS thresholds, has been described elsewhere (Ap et al., 2025).

### Body mass and body composition

Body mass (BM) and body composition were assessed under fasting conditions, with participants wearing only underwear. Daily BM was measured using a calibrated supine scale specifically designed for accurate weighing in the horizontal position. Body composition was determined by dual‐energy X‐ray absorptiometry (DXA; Hologic QDR‐4500C, APEX software version 5.6.1.7, Hologic Inc., Marlborough, MA, USA) at BDC‐2, HDT16, HDT32, HDT44, HDT56 and during recovery (R+3 and R+11). To ensure high reproducibility, participants were repositioned according to standardized guidelines, using previous scans as visual references. Scans were analysed using the Classic calibration mode, which provides more accurate body composition estimates than the NHANES calibration (Ng et al., 2018; Smith‐Ryan et al., 2017).

All DXA data were processed by the same trained physician and independently reviewed by a second assessor. Regions of interest (ROIs) were automatically defined by the APEX software and subsequently verified or manually refined when required. The Hologic system provided estimates of fat mass (FM), FFM and regional composition. Regional ROIs included whole‐body, lower‐body and upper‐body. The upper‐body region encompassed both trunk and arms; the trunk was also analysed separately as a subregion of the upper‐body. Consistent with previous reports, the sum of FM and FFM differed from independently measured total BM by ∼1 kg, probably due to internal rounding, segmentation and calibration algorithms (Genton et al., 2006; Prior et al., 1997); therefore, analyses relied on measured BM rather than DXA‐derived totals.

Total and regional (lower‐body, upper‐body and trunk) FM and FFM were derived from these measures. Inter‐individual differences in stature were addressed by calculating BMI (BM·height^−2^), FFM index (FFMI = FFM·height^−2^) and FM index (FMI = FM·height^−2^), with analogous indices for segmental compartments. Because the HDT32 assessment was performed immediately after a 24 h fast, these data were excluded from descriptive and inferential analyses to avoid bias from fasting‐induced shifts in hydration and tissue composition.

### Magnetic resonance imaging of body composition

Magnetic resonance imaging (MRI) was used to assess fat distribution, skeletal muscle composition, and total lean and fat tissue volumes before (BDC‐9) and during bed rest (HDT52). The original acquisition and analysis of these data have been published previously (Mandic et al., 2025), where detailed methodological information can be found. In brief, imaging was performed in the supine position using a 1.5 T scanner (Siemens Sola, Siemens Healthcare, Erlangen, Germany) with a two‐point Dixon fat–water sequence. Fat and muscle segmentation were carried out using AMRA Researcher software (AMRA Medical AB, Linköping, Sweden) (Mandic et al., 2020). MRI‐derived variables included total lean tissue volume (TLT), visceral adipose tissue (VAT), subcutaneous abdominal adipose tissue (SAT), total thigh fat‐free muscle volume, individual thigh muscle volumes (left/right, anterior/posterior), muscle fat infiltration, liver proton density fat fraction, total adipose tissue (TAT) and the fat ratio (TAT/TLT).

### RMR and substrate utilization

RMR and substrate oxidation were assessed at BDC‐14, BDC‐1 and HDT60 by indirect calorimetry (Quark RMR, Cosmed, Rome, Italy). Measurements were performed in the morning after an overnight fast, approximately 1 h after awakening, with participants remaining in the supine position. After a 10 min acclimatization period, ${\dot{V}}_{O_{2}}$ and carbon dioxide production (${\dot{V}}_{CO_{2}}$) were continuously recorded for 20 min using a large ventilated‐canopy system. The calorimeter was calibrated before each session according to manufacturer instructions.

To ensure consistency across sessions and devices, a simulation‐based calibration protocol was performed using an external mixing chamber. Compressed reference gas was blended with ambient air under controlled conditions via a precision mass flow controller. The mixed gas was then aspirated by the device pump at fixed ventilation rates (25 and 45 L min^−^^1^), thereby reproducing subject‐like canopy measurement conditions without altering the flow dynamics of the system. Eight simulation points (four per ventilation level) spanning physiologically relevant $F_{EO_{2}}$ and $F_{\mathrm{EC}O_{2}}$ values were generated. Theoretical ${\dot{V}}_{O_{2}}$ and ${\dot{V}}_{CO_{2}}$ values were calculated from known gas fractions and flow rates, and regression‐derived correction factors were applied to measured data.

Daily protein oxidation was estimated from 24 h urinary nitrogen excretion. Total nitrogen was quantified using the Dumas combustion method (De Preter et al., 2007) in a continuous‐flow elemental analyser coupled to an isotope‐ratio mass spectrometer (ANCA‐2020, Europa Scientific, Crewe, UK). Energy expenditure was calculated using Weir's equation (Weir, 1949), and the respiratory quotient (RQ) defined as ${\dot{V}}_{CO_{2}}/{\dot{V}}_{O_{2}}$. Non‐protein RQ (NPRQ) and corresponding carbohydrate and fat oxidation rates were computed using Frayn's equations, incorporating ${\dot{V}}_{O_{2}}$, ${\dot{V}}_{CO_{2}}$ and urinary nitrogen excretion (Ferrannini, 1988; Frayn, 1983). One calorimetry measurement at BDC‐1 was excluded due to a technical issue affecting data reliability.

Resting heart rate (HR, min^−1^), systolic and diastolic blood pressure (SBP, DBP; mmHg), mean arterial pressure (MAP), and tympanic temperature (Tty,°C) were measured each morning upon awakening and in the fasted supine state using calibrated automated devices (automated sphygmomanometer, Dinamap^®^ Carescape V100, GE HealthCare, Chicago, IL, USA; tympanic thermometer, Genius 3, Covidien, Dublin, Ireland).

### Isometric muscle strength

Maximal voluntary isometric contractions (MVCs) were recorded unilaterally (left side) using an isokinetic dynamometer (CON‐TREX AG, Dübendorf, Switzerland) for knee extensors and flexors, as well ankle plantar flexors (ankle extension) and dorsiflexors (ankle flexion). For clarity, ankle plantar flexion corresponds to ankle extension, and ankle dorsiflexion corresponds to ankle flexion. Participants were familiarized with the equipment and testing procedures prior to the measurements. As part of the ESA standard measurements, MVCs were obtained once during the baseline phase (BDC‐4) and once after bed rest (R+2). Knee extension and flexion MVCs were assessed in a seated position with the hip and knee joints fixed at 90° and 80° of flexion, respectively. Ankle extension and flexion MVCs were performed in the prone position, with the ankle joints maintained at 0° plantar flexion, corresponding to a right angle between the sole of the foot and the tibia. The test protocol was identical for each muscle group. After a standardized warm‐up consisting of 5 mins of cycling at 50 W in a neutral position, participants were instructed to perform a maximum extension contraction, followed 30 s later by a maximum flexion contraction. After an additional 30 s, pairs of extension and flexion contractions were repeated until three complete sets of extension/flexion contractions had been recorded. Each contraction lasted 5–7 s. Rest intervals of 2 mins between repetitions and 5 mins between tests were provided to minimize fatigue. Force signals from the dynamometer load cells were amplified (Kistler 5006), sampled at 1 kHz, and stored for subsequent offline analysis. Data processing included correction of signal offsets and extraction of the highest force value achieved in each trial. For analysis, both the peak and the force average over a 0.5 s window centred on the peak were extracted. The 0.5 s averaging window was chosen to provide a robust estimate of sustained maximal force while minimizing the influence of transient fluctuations and signal noise, in line with previous work (Rodrigues et al., 2023; Sara et al., 2023).

### Jumping test

Jumping capacity was assessed through three maximal countermovement jumps (CMJs) performed on a force platform (Leonardo GRFP, Novotec Medical GmbH, Pforzheim, Germany) on BDC‐3 and R+0, as one of the first tests upon standing, as reported elsewhere (Kramer et al., 2021). Before testing, all participants were instructed to perform a series of three squats and practise the correct CMJ technique. During each attempt, they placed their hands on their hips, rapidly descended to a half‐squat position and immediately performed a maximal vertical jump. A 1 min rest interval was provided between jumps. Vertical ground reaction forces were recorded via a data acquisition system (Power1401‐3, CED, Cambridge, UK). Jump height was calculated from the velocity at take‐off (anti‐derivative of the vertical ground reaction force) using the equation: jump height = (velocity at take‐off)^2^/2 *g*. Jumping power was computed as the product of vertical ground reaction force and velocity. The highest jump achieved by each participant was retained for analysis.

### Statistical methods

#### Descriptive analyses

Baseline characteristics and raw outcome values were summarized by group using means ± SD. Because minor baseline differences in BM were present despite randomization, all composition variables were normalized to height^2^ (BMI, FFMI, FMI) and these indices were used in all inferential analyses. Daily BMI and temperature time series were smoothed using 3 day and 5 day moving averages, respectively.

#### Outcomes

Four domains were considered as primary outcomes: (i) vital parameters, (ii) body composition (total and regional FFMI, total FMI from DXA), (iii) resting metabolism (RMR, RQ, carbohydrate and fat utilization) and (iv) neuromuscular function (MVC of knee and ankle plantar; CMJ height, force, power, take‐off velocity). In addition, maximal cycling power derived from the incremental upright cardiopulmonary exercise test used to determine ${\dot{V}}_{O_{2}\mathrm{peak}}$ was analysed as a modality‐specific performance outcome. Depending on the measurement schedule, analyses used both BDC and either end‐HDT (RMR, vital parameters) or first recovery values (neuromuscular function and maximal cycling power), or BDC, multiple HDT points and first recovery values (DXA).

#### Linear mixed‐effects models

Changes in outcomes were analysed using linear mixed‐effects models (LMMs) including all available repeated measures from the last BDC through HDT and the first recovery assessment. Fixed effects were group (C, EX, EX‐AG), time (categorical) and group‐by‐time. A subject‐specific random intercept accounted for within‐participant correlation. The within‐subject covariance structure was selected using the Bayesian Information Criterion (generally compound symmetry), with group‐specific residual variances allowed when appropriate. Kenward–Roger degrees of freedom were used. Model assumptions were assessed using standardized residuals and Q–Q plots.

The primary inferential objective was to test whether EX or EX‐AG attenuated bed rest‐induced changes relative to C at the prespecified main endpoint: first recovery for body composition and neuromuscular outcomes; and end‐HDT for RMR and vital parameters. When both time points existed (body composition), the alternative time (typically end‐HDT) was considered secondary. A pre‐specified endpoint‐specific contrast was computed at each main endpoint to test overall between‐group differences in change from baseline (global ΔΔ test). Pairwise ΔΔ contrasts (EX *vs*. C, EX‐AG *vs*. C, EX‐AG *vs*. EX) were then derived from the same model. Least‐squares means for within‐group changes (Δ) and corresponding ΔΔ estimates were reported only at these targeted endpoints, with 95% confidence intervals.

For RMR and substrate‐utilization outcomes, additional models included concurrent FFM to evaluate whether group differences persisted after accounting for tissue‐level changes. Model comparisons relied on likelihood‐ratio tests and the Akaike Information Criterion (AIC).

#### Modelling the RMR–FFM relationship

To quantify how much of the change in RMR was explained by changes in FFM, two complementary analyses were performed. First, the association between ΔRMR and ΔFFM was assessed using simple regression. Second, a longitudinal LMM was fitted with RMR (BDC, HDT) as outcome and repeated FFM, time, group and group‐by‐time as fixed effects; the time‐by‐FFM interaction was tested and removed as it was non‐significant. This model estimated group‐specific vertical shifts in RMR between BDC and HDT at a common FFM, with corresponding 95% confidence intervals and pairwise contrasts.

#### Multivariate analyses

Beyond univariate modelling, multivariate patterns of broader physiological adaptation were explored. A principal component analysis (PCA) was first performed on all Δ‐variables to characterize coordinated physiological changes across domains. PCA was conducted on z‐standardized data and interpreted using eigenvalues, scree‐plot inspection and variable contributions. To investigate multivariate associations with ΔRMR, a sparse partial least squares (sPLS) regression model was fitted and a parsimonious model with one latent component and ten selected predictors was specified. Model performance was assessed using the correlation between observed and predicted ΔRMR, and through 6‐fold cross‐validation procedure (Stone, 1974) to estimate predictive generalization.

#### Presentation of results

Raw values are presented as mean ± SD; model‐based estimates (Δ, ΔΔ) are reported as least‐squares means with corresponding 95% confidence intervals (95% CIs) derived from the mixed‐effects models. All mixed‐effects modelling was performed in SAS 9.4 (SAS Institute Inc., Cary, NC, USA) using restricted maximum likelihood (REML). Multivariate analyses (PCA, sPLS) were performed in R 4.5.1. All tests were two‐sided. The significance threshold was α = 0.05 for main effects and pairwise comparisons, and α = 0.10 for group‐by‐time interactions and prespecified endpoint‐specific overall contrasts. No multiplicity correction was applied.

## Results

### Resting vital parameters remain largely stable, with exercise mitigating increases in blood pressure

Changes in resting vital parameters between BDC‐1 and HDT60 are summarized in Table 1. Core body temperature remained stable across all phases, with no significant main or interaction effects (*P* > 0.25). Heart rate increased in C (+8.7 bpm, 95% CI 1.1–16.3), whereas both exercise interventions largely maintained pre‐bed rest levels (EX +2.0 bpm; EX‐AG +1.4 bpm). However, the overall time effect did not reach statistical significance (*P* = 0.069), and no group‐by‐time interaction was observed (*P = *0.314). Changes in resting blood pressure exhibited clearer group differences. DBP increased significantly in C (+11.40 mmHg, 95% CI 5.86–16.90), whereas changes were minimal in both intervention groups (EX: +3.31 mmHg; EX‐AG: +2.56 mmHg) resulting in significant between‐group contrasts (EX *vs*. C: −8.06 mmHg, *P* = 0.043; EX‐AG *vs*. C: −8.81 mmHg, *P* = 0.029). MAP and SBP followed similar patterns, with significant time effects (*P* = 0.004 and 0.015, respectively) but no significant group‐by‐time interactions (*P* > 0.10). Overall, both countermeasure protocols attenuated the rise in resting blood pressure observed under bed rest, while heart rate and body temperature remained unchanged.

**Table 1:** Vital values by group, and comparison at HDT60 *vs*. baseline (BDC‐1)

Variable	Group	BDC‐1	HDT60	ΔHDT60	ΔΔHDT60	Global time *P*	Global group *P*	Global interaction *P*
Tty (°C)^*^	C	36.3 (0.2)	36.4 (0.2)	0.08 (−0.14; 0.29)	EX *vs*. C: 0.04 (−0.27; 0.35); *P* = 0.782	0.256	0.686	0.813
	EX	36.2 (0.3)	36.3 (0.3)	0.12 (−0.10; 0.34)	EX‐AG *vs*. C: −0.05 (−0.36; 0.25); *P* = 0.719			
	EX‐AG	36.3 (0.4)	36.4 (0.3)	0.02 (−0.20; 0.24)	EX‐AG *vs*. EX: −0.10 (−0.40; 0.21); *P* = 0.526			
**HR (min^−1^)**	C	61.8 (10.4)	70.5 (11.9)	8.69 (1.09; 16.3)	EX *vs*. C: −6.69 (−17.4; 4.05); *P* = 0.209	0.069	0.037	0.315
	EX	56.3 (3.0)	58.3 (9.1)	2.00 (−5.59; 9.59)	EX‐AG *vs*. C: −7.25 (−18.0; 3.49); *P* = 0.175			
	EX‐AG	56.8 (8.3)	58.3 (8.1)	1.44 (−6.16; 9.03)	EX‐AG *vs*. EX: −0.56 (−11.3; 10.2); *P* = 0.914			
**SBP (mmHg)**	C	114 (8)	120 (13)	6.50 (−0.06; 13.1)	EX *vs*. C: −4.75 (−14.0; 4.53); *P* = 0.299	0.015	0.114	0.499
	EX	113 (5)	115 (8)	1.75 (−4.81; 8.31)	EX‐AG *vs*. C: −0.25 (−9.53; 9.03); *P* = 0.956			
	EX‐AG	107 (3)	113 (7)	6.25 (−0.31; 12.8)	EX‐AG *vs*. EX: 4.50 (−4.78; 13.8); *P* = 0.325			
**DBP (mmHg)**	C	60.4 (8.0)	71.8 (11.2)	11.4 (5.86; 16.9)	EX *vs*. C: −8.06 (−15.9; −0.26); *P* = 0.043	0.001	0.139	0.053
	EX	60.4 (4.3)	63.7 (7.3)	3.31 (−2.20; 8.83)	EX‐AG *vs*. C: −8.81 (−16.6; −1.01); *P* = 0.029			
	EX‐AG	58.3 (4.0)	60.9 (6.9)	2.56 (−2.95; 8.08)	EX‐AG *vs*. EX: −0.75 (−8.55; 7.05); *P* = 0.843			
**MAP (mmHg)**	C	81.4 (8.1)	89.4 (11.7)	8.00 (2.29; 13.7)	EX *vs*. C: −4.88 (−12.9; 3.19); *P* = 0.223	0.004	0.102	0.441
	EX	80.0 (3.7)	83.1 (6.8)	3.13 (−2.58; 8.83)	EX‐AG *vs*. C: −3.63 (−11.7; 4.44); *P* = 0.361			
	EX‐AG	76.4 (2.5)	80.8 (6.4)	4.38 (−1.33; 10.1)	EX‐AG *vs*. EX: 1.25 (−6.82; 9.32); *P* = 0.751			

Values are mean ± SD.

^*^Moving average over five points.

Δ and ΔΔ represent within‐group changes and between‐group differences in change from baseline, respectively, estimated from mixed models, with corresponding 95% confidence intervals. Global *P* values correspond to Type III tests of fixed effects from the mixed model.

Abbreviations: BDC, baseline period; C, control group; DBP, diastolic blood pressure; EX, exercise‐only group; EX‐AG, exercise plus artificial gravity group; HDT, head‐down tilt bed rest; HR, heart rate; MAP, mean arterial pressure; SBP, systolic blood pressure; Tty, tympanic temperature.

### Combined exercise and AG effectively preserve lower body lean mass

Changes in BM and DXA‐derived composition are summarized in Tables 2 and 3. Total BM and BMI decreased significantly during bed rest across all groups (mean reductions of ≈2 kg and 0.65 kg m^−^^2^ respectively by HDT56; *P* < 0.001). The pre‐specified endpoint contrast at HDT56 revealed between‐group differences in changes from baseline. At this time, EX‐AG showed a protective effect compared with C at HDT56 with a smaller reduction in BMI (0.27 kg m^−2^, *P = *0.033), whereas no between‐group differences were observed at R+3.

**Table 2:** DXA values by group, and comparison at R+3 *vs*. baseline (BDC‐2)

Variable	Group	BDC‐2	HDT16	HDT44	HDT56	R+3	ΔR+3	ΔΔR+3	R+3 contrast *P*	Global time *P*	Global group *P*	Global interaction *P*
BMI* (kg m^−2^)	C	24.1 (1.5)	23.4 (1.6)	23.2 (1.6)	23.4 (1.7)	23.7 (1.7)	−0.41 (−0.59; −0.24)	EX *vs*. C: 0.11 (−0.14; 0.35); *P* = 0.377	0.253	<0.001	0.814	0.565
	EX	23.8 (2.1)	23.3 (2.0)	23.1 (1.9)	23.3 (1.9)	23.5 (1.9)	−0.30 (−0.48; −0.13)	EX‐AG *vs*. C: 0.21 (−0.04; 0.45); *P* = 0.098				
	EX‐AG	23.4 (1.9)	22.9 (1.8)	22.8 (1.8)	22.8 (1.8)	23.2 (1.8)	−0.21 (−0.38; −0.04)	EX‐AG *vs*. EX: 0.10 (−0.15; 0.34); *P* = 0.435				
**FFMI (kg m^−2^)**	C	18.3 (1.2)	17.8 (1.1)	17.6 (1.2)	17.5 (1.1)	18.0 (1.2)	−0.39 (−0.56; −0.22)	EX *vs*. C: −0.02 (−0.26; 0.22); *P* = 0.898	0.005	<0.001	0.674	0.036
	EX	18.6 (1.1)	18.2 (1.0)	18.0 (0.9)	17.9 (0.9)	18.2 (1.0)	−0.40 (−0.57; −0.23)	EX‐AG *vs*. C: 0.34 (0.10; 0.58); *P* = 0.006				
	EX‐AG	18.0 (1.2)	17.7 (1.1)	17.5 (1.1)	17.5 (1.0)	17.9 (1.0)	−0.04 (−0.21; 0.13)	EX‐AG *vs*. EX: 0.36 (0.12; 0.60); *P* = 0.004				
**FMI (kg m^−2^)**	C	5.33 (1.05)	5.35 (1.08)	5.29 (1.10)	5.41 (1.00)	5.34 (1.18)	0.01 (−0.11; 0.14)	EX *vs*. C: 0.07 (−0.12; 0.25); *P* = 0.475	0.107	0.008	0.651	0.296
	EX	4.85 (1.11)	4.74 (1.10)	4.81 (1.07)	4.95 (1.12)	4.93 (1.01)	0.08 (−0.05; 0.21)	EX‐AG *vs*. C: −0.13 (−0.31; 0.05); *P* = 0.168				
	EX‐AG	4.99 (1.27)	4.85 (1.30)	4.90 (1.18)	4.97 (1.27)	4.88 (1.27)	−0.11 (−0.24; 0.01)	EX‐AG *vs*. EX: −0.19 (−0.37; −0.01); *P* = 0.038				
**Lower‐body FFMI (kg m^−2^)**	C	6.01 (0.31)	5.72 (0.35)	5.49 (0.42)	5.44 (0.38)	5.61 (0.37)	−0.40 (−0.48; −0.32)	EX *vs*. C: 0.12 (0.01; 0.23); *P* = 0.037	<0.001	<0.001	0.477	<0.001
	EX	6.09 (0.48)	5.93 (0.42)	5.76 (0.40)	5.67 (0.40)	5.81 (0.45)	−0.28 (−0.36; −0.20)	EX‐AG *vs*. C: 0.23 (0.11; 0.34); *P* < 0.001				
	EX‐AG	5.83 (0.36)	5.68 (0.36)	5.55 (0.36)	5.51 (0.35)	5.66 (0.33)	−0.17 (−0.25; −0.09)	EX‐AG *vs*. EX: 0.11 (−0.01; 0.22); *P* = 0.064				
**Upper‐body FFMI (kg m^−2^)**	C	11.1 (1.0)	10.8 (0.8)	10.80 (0.9)	10.7 (0.8)	11.0 (0.9)	−0.02 (−0.16; 0.11)	EX *vs*. C: −0.13 (−0.32; 0.06); *P* = 0.185	0.056	<0.001	0.819	0.477
	EX	11.3 (0.6)	11.0 (0.6)	10.9 (0.5)	10.9 (0.5)	11.1 (0.6)	−0.15 (−0.29; −0.02)	EX‐AG *vs*. C: 0.11 (−0.09; 0.30); *P* = 0.275				
	EX‐AG	10.9 (0.9)	10.8 (0.8)	10.7 (0.8)	10.7 (0.7)	11 (0.8)	0.08 (−0.05; 0.22)	EX‐AG *vs*. EX: 0.24 (0.04; 0.43); *P* = 0.017				
**Trunk FFMI (kg m^−2^)**	C	8.6 (0.7)	8.4 (0.6)	8.4 (0.6)	8.3 (0.5)	8.5 (0.6)	−0.07 (−0.20; 0.05)	EX *vs*. C: −0.06 (−0.24; 0.12); *P* = 0.489	0.046	<0.001	0.792	0.220
	EX	8.8 (0.5)	8.6 (0.5)	8.4 (0.5)	8.5 (0.5)	8.6 (0.5)	−0.13 (−0.26; −0.01)	EX‐AG *vs*. C: 0.16 (−0.02; 0.33); *P* = 0.083				
	EX‐AG	8.5 (0.6)	8.4 (0.6)	8.3 (0.5)	8.3 (0.6)	8.5 (0.6)	0.08 (−0.04; 0.21)	EX‐AG *vs*. EX: 0.22 (0.04; 0.39); *P* = 0.016				

Values are mean ± SD.

^*^Moving average over three points.

Δ and ΔΔ represent within‐group changes and between‐group differences in change from baseline, respectively, estimated from mixed models using all time points, with corresponding 95% confidence intervals. R+3 contrast *P* refers to the pre‐specified endpoint‐specific contrast testing the overall between‐group difference in change from baseline. Global *P* values correspond to Type III tests of fixed effects from the mixed model.

Abbreviations: BDC, baseline period; BMI, body mass index; C, control group; DXA, dual‐energy X‐ray absorptiometry; EX, exercise‐only group; EX‐AG, exercise plus artificial gravity group; FFMI, fat‐free mass index; FMI, fat mass index; HDT, head‐down tilt bed rest; R, recovery.

**Table 3:** DXA comparison at HDT56 *vs*. baseline (BDC‐2)

Variable	Group	ΔHDT56	ΔΔHDT56	HDT56 contrast *P*
BMI* **(kg m^−2^)**	C	−0.80 (−0.98; −0.63)	EX *vs*. C: 0.17 (−0.08; 0.41); *P* = 0.183	0.098
	EX	−0.64 (−0.81; −0.46)	EX‐AG *vs*. C: 0.27 (0.02; 0.51); *P* = 0.033	
	EX‐AG	−0.54 (−0.71; −0.36)	EX‐AG *vs*. EX: 0.10 (−0.14; 0.35); *P* = 0.412	
**FFMI (kg m^−2^)**	C	−0.88 (−1.05; −0.71)	EX *vs*. C: 0.16 (−0.08; 0.40); *P* = 0.189	0.01
	EX	−0.72 (−0.89; −0.55)	EX‐AG *vs*. C: 0.37 (0.13; 0.61); *P* = 0.003	
	EX‐AG	−0.51 (−0.68; −0.34)	EX‐AG *vs*. EX: 0.21 (−0.03; 0.45); *P* = 0.081	
**FMI (kg m^−2^)**	C	0.09 (−0.04; 0.21)	EX *vs*. C: 0.01 (−0.17; 0.19); *P* = 0.921	0.334
	EX	0.10 (−0.03; 0.22)	EX‐AG *vs*. C: −0.11 (−0.29; 0.07); *P* = 0.219	
	EX‐AG	−0.03 (−0.15; 0.10)	EX‐AG *vs*. EX: −0.12 (−0.30; 0.06); *P* = 0.184	
**Lower‐body FFMI (kg m^−2^)**	C	−0.57 (−0.65; −0.49)	EX *vs*. C: 0.15 (0.03; 0.26); *P* = 0.011	<0.001
	EX	−0.42 (−0.50; −0.34)	EX‐AG *vs*. C: 0.25 (0.13; 0.36); *P* < 0.001	
	EX‐AG	−0.32 (−0.40; −0.24)	EX‐AG *vs*. EX: 0.10 (−0.01; 0.21); *P* = 0.086	
**Upper‐body FFMI (kg m^−2^)**	C	−0.33 (−0.47; −0.20)	EX *vs*. C: −0.01 (−0.20; 0.18); *P* = 0.905	0.297
	EX	−0.34 (−0.48; −0.21)	EX‐AG *vs*. C: 0.13 (−0.07; 0.32); *P* = 0.199	
	EX‐AG	−0.21 (−0.34; −0.07)	EX‐AG *vs*. EX: 0.14 (−0.06; 0.33); *P* = 0.161	
**Trunk FFMI (kg m^−2^)**	C	−0.32 (−0.45; −0.20)	EX *vs*. C: 0.06 (−0.12; 0.23); *P* = 0.527	0.084
	EX	−0.26 (−0.39; −0.14)	EX‐AG *vs*. C: 0.19 (0.02; 0.37); *P* = 0.031	
	EX‐AG	−0.13 (−0.25; 0.00)	EX‐AG *vs*. EX: 0.14 (−0.04; 0.32); *P* = 0.123	

^*^Moving average over three points.

Δ and ΔΔ represent within‐group changes and between‐group differences in change from baseline, respectively, estimated from mixed models using all time points, with corresponding 95% confidence intervals. HDT56 contrast *P* refers to the pre‐specified endpoint‐specific contrast testing the overall between‐group difference in change from baseline.

Abbreviations: BDC, baseline period; BMI, body mass index; C, control group; DXA, dual‐energy X‐ray absorptiometry; EX, exercise‐only group; EX‐AG, exercise plus artificial gravity group; FFMI, fat‐free mass index; FMI, fat mass index; HDT, head‐down tilt bed rest.

FFMI declined markedly over time, particularly in the lower body, where the magnitude of loss differed between groups (group‐by‐time interaction *P* ≤ 0.001). The largest reductions occurred in the control group (−0.57 kg m^−2^ at HDT56 and −0.40 kg m^−2^ at R+3), whereas both exercise interventions attenuated this loss (EX −0.42 kg m^−2^ and −0.28 kg m^−2^; EX‐AG −0.32 kg m^−2^ and −0.17 kg m^−2^). Endpoint‐specific contrasts confirmed significantly greater preservation with EX‐AG *versus* C at both HDT56 and R+3 (ΔΔ = +0.25 kg m^−2^ and +0.23 kg m^−2^, both *P* < 0.001), with smaller but consistent effects in EX (ΔΔ = +0.15 kg m^−2^, *P* = 0.011 and +0.12 kg m^−2^, *P* = 0.037). When compared with EX, the effect size under EX‐AG was larger but did not reach significance (ΔΔ = +0.10 kg m^−2^, *P = *0.086 at HDT56; +0.11 kg m^−2^, *P = *0.064 at R+3). A similar pattern was observed for trunk FFMI, which was better preserved in EX‐AG than in C (ΔΔ = +0.19 kg m^−2^ at HDT56, *P = *0.031), while the magnitude of upper‐body FFMI loss did not differ significantly between groups.

Total FMI showed no consistent changes during HDT. At R+3, EX‐AG exhibited a modest decrease, although the endpoint‐specific contrast did not reach significance (*P* ≈ 0.10). While regional FMI was not a predefined primary outcome, the endpoint‐specific contrast at R+3 indicated overall between‐group differences for upper‐body FMI (*P = *0.028) and a similar, though weaker, effect for trunk FMI (*P = *0.069). Pairwise contrasts confirmed that this former finding was attributable to a modest reduction from baseline in EX‐AG compared with both C (−0.14 kg m^−2^, *P = *0.027) and EX (−0.16 kg m^−2^, *P* = 0.016) at this time point.

Overall, both exercise countermeasures attenuated lean tissue loss, with EX‐AG providing the greatest preservation, particularly in the lower body. These results are consistent with and reinforced recently published MRI‐derived data (Mandic et al., 2025).

### Combined exercise and AG preserve RMR

Despite differences in total caloric intake driven by exercise‐related energy compensation, macronutrient distribution remained comparable between groups throughout bed rest (Table 4), with protein intake maintained at ∼1.15–1.2 g kg^−^^1^ day^−^^1^, fat contributing ∼35% of total energy intake, and carbohydrate ∼49.5–51.5%.

**Table 4:** Energy intake and macronutrient distribution across study phases

Variable	Time period	C (*n* = 8)	EX (*n* = 8)	EX‐AG (*n* = 8)	All (*n* = 24)
**Energy intake (PAL)**	BDC‐14–BDC‐1	1.60 (0.008)	1.61 (0.009)	1.61 (0.007)	1.61 (0.008)
	HDT1–HDT10	1.30 (0.013)	1.45 (0.017)	1.45 (0.016)	1.40 (0.071)
	HDT11–HDT49	1.44 (0.065)	1.64 (0.082)	1.64 (0.103)	1.57 (0.126)
	HDT50–HDT60	1.49 (0.007)	1.65 (0.018)	1.65 (0.019)	1.60 (0.078)
	R+0–R+13	1.60 (0.000)	1.60 (0.002)	1.60 (0.004)	1.60 (0.002)
**Energy intake (kcal day^−1^)**	BDC‐14–BDC‐1	2651.5 (289.8)	2707.7 (316.4)	2620.0 (214.9)	2659.8 (267.3)
	HDT1–HDT10	2156.6 (233.7)	2434.2 (264.8)	2370.8 (211.3)	2320.6 (257.4)
	HDT11–HDT49	2383.1 (295.6)	2750.7 (257.4)	2677.6 (261.9)	2603.8 (306.5)
	HDT50–HDT60	2462.5 (280.8)	2769.6 (303.5)	2693.6 (240.6)	2641.9 (295.7)
	R+0–R+13	2646.7 (300.3)	2696.9 (324.4)	2610.1 (219.8)	2651.3 (274.8)
**Carbohydrate (%)**	BDC‐14–BDC‐1	50.7 (0.96)	50.7 (1.03)	50.7 (1.43)	50.7 (1.10)
	HDT1–HDT10	49.3 (1.24)	50.1 (0.95)	50.2 (1.29)	49.8 (1.18)
	HDT11–HDT49	49.7 (1.85)	50.4 (1.46)	50.0 (1.66)	50.0 (1.61)
	HDT50–HDT60	50.1 (1.11)	50.7 (1.31)	50.9 (1.48)	50.6 (1.30)
	R+0–R+13	51.6 (1.19)	50.8 (1.54)	50.7 (1.61)	51.0 (1.44)
**Fat (% energy)**	BDC‐14–BDC‐1	36.0 (0.53)	35.7 (0.56)	35.7 (0.43)	35.8 (0.51)
	HDT1–HDT10	35.4 (0.46)	35.2 (0.32)	35.2 (0.26)	35.3 (0.34)
	HDT11–HDT49	35.4 (1.69)	35.5 (0.94)	36.0 (1.52)	35.6 (1.39)
	HDT50–HDT60	35.0 (0.42)	35.1 (0.35)	34.9 (0.47)	35.0 (0.40)
	R+0–R+13	34.7 (0.35)	34.9 (0.33)	34.9 (0.36)	34.8 (0.35)
**Protein (g kg^−^^1^ day^−^^1^)**	BDC‐14–BDC‐1	1.16 (0.04)	1.15 (0.05)	1.15 (0.05)	1.16 (0.04)
	HDT1–HDT10	1.09 (0.06)	1.11 (0.05)	1.12 (0.06)	1.11 (0.06)
	HDT11–HDT49	1.17 (0.05)	1.21 (0.06)	1.21 (0.06)	1.20 (0.07)
	HDT50–HDT60	1.21 (0.03)	1.22 (0.02)	1.23 (0.02)	1.22 (0.02)
	R+0–R+13	1.20 (0.01)	1.20 (0.01)	1.20 (0.01)	1.20 (0.01)

Values are mean ± SD.

Exercise‐related caloric compensation was applied to EX and EX‐AG based on estimated training energy expenditure.

Abbreviations: BDC, baseline period; C, control group; EX, exercise‐only group; EX‐AG, exercise plus artificial gravity group; HDT, head‐down tilt bed rest; R, recovery.

Changes in RMR between BDC‐1 and HDT60 are summarized in Table 5, with FFM‐adjusted results presented in Table 6. RMR responses during bed rest differed between groups. RMR declined markedly in the control group (–157 kcal 24 h^−^^1^), whereas both exercise interventions attenuated this decline (group‐by‐time interaction *P* = 0.029). The EX‐group showed an attenuated decrease (–121 kcal·24 h^−^^1^), while EX‐AG largely maintained pre–bed rest levels (−23 kcal·24 h^−^^1^). Pairwise contrasts confirmed a significant preservation of RMR in EX‐AG compared with C (ΔΔ = +133 kcal·24 h^−^^1^, *P = *0.017), whereas the difference between EX and C was not statistically significant (ΔΔ = +36 kcal·24 h^−^^1^, *P = *0.575). Overall, EX‐AG was the only intervention that effectively prevented the decline in RMR.

**Table 5:** Indirect calorimetry values by group, and comparison at HDT60 *vs*. baseline (BDC‐1)

Variable	Group	BDC‐1	HDT60	ΔHDT60	ΔΔHDT60	Global time *P*	Global group *P*	Global interaction *P*
**RMR (kcal 24 h^−1^)**	C	1626 (191)	1470 (149)	−157 (−256; −57.3)	EX *vs*. C: 35.8 (−98.1; 170); *P* = 0.575)	<0.001	0.683	0.029
	EX	1682 (181)	1561 (173)	−121 (−230; −11.5)	EX‐AG *vs*. C: 133 (29.6; 237); *P* = 0.017			
	EX‐AG	1575 (123)	1568 (127)	−23.3 (−71.8; 25.2)	EX‐AG *vs*. EX: 97.4 (−15.5; 210); *P* = 0.083			
**RQ**	C	0.83 (0.03)	0.86 (0.04)	0.04 (0.01; 0.06)	EX *vs*. C: 0.00 (−0.03; 0.03); *P* = 0.966	<0.001	0.070	0.087
	EX	0.80 (0.03)	0.84 (0.02)	0.04 (0.01; 0.06)	EX‐AG *vs*. C: −0.03 (−0.06; 0.00); *P* = 0.051			
	EX‐AG	0.81 (0.03)	0.81 (0.04)	0.00 (−0.02; 0.03)	EX‐AG *vs*. EX: −0.03 (−0.06; 0.00); *P* = 0.055			
**Carbohydrate utilization (mg min^−1^)**	C	111 (31)	130 (36)	19.4 (1.65; 37.1)	EX *vs*. C: 7.09 (−18.0; 32.2); *P* = 0.561	0.006	0.125	0.121
	EX	87.3 (31.8)	114 (25)	26.5 (8.74; 44.2)	EX‐AG *vs*. C: −19.1 (−44.9; 6.74); *P* = 0.139			
	EX‐AG	85.3 (27.4)	89.7 (34.6)	0.29 (−18.5; 19.1)	EX‐AG *vs*. EX: −26.2 (−52.0; −0.35); *P* = 0.047			
**Fat utilization (mg min^−1^)**	C	56.8 (18.5)	34.7 (17.0)	−22.1 (−33.4; −10.8)	EX *vs*. C: 0.52 (−15.5; 16.5); *P* = 0.946	<0.001	0.156	0.054
	EX	68.1 (17.8)	46.6 (12.8)	−21.6 (−32.9; −10.3)	EX‐AG *vs*. C: 18.2 (1.76; 34.6); *P* = 0.032			
	EX‐AG	61.4 (13.2)	57.1 (16.8)	−3.90 (−15.8; 8.02)	EX‐AG *vs*. EX: 17.7 (1.24; 34.1); *P* = 0.036			
**Protein utilization (mg min^−1^)**	C	42.8 (8.1)	49.9 (7.3)	7.08 (0.99; 13.2)	EX *vs*. C: −1.66 (−10.3; 6.95); *P* = 0.692	0.003	0.648	0.881
	EX	47.5 (13.1)	52.9 (11.9)	5.42 (−0.67; 11.5)	EX‐AG *vs*. C: −1.96 (−10.8; 6.89); *P* = 0.649			
	EX‐AG	46.8 (7.1)	51.7 (5.8)	5.11 (−1.32; 11.5)	EX‐AG *vs*. EX: −0.30 (−9.16; 8.55); *P* = 0.944			
**Urinary nitrogen excretion (g day^−1^**)	C	9.87 (1.86)	11.5 (1.7)	1.63 (0.20; 3.06)	EX *vs*. C: −0.38 (−2.41; 1.64); *P* = 0.698	0.005	0.610	0.743
	EX	10.9 (3.0)	12.2 (2.7)	1.25 (−0.18; 2.68)	EX‐AG *vs*. C: −0.76 (−2.78; 1.27); *P* = 0.446			
	EX‐AG	11.0 (1.7)	11.9 (1.3)	0.88 (−0.55; 2.31)	EX‐AG *vs*. EX: −0.37 (−2.40; 1.65); *P* = 0.706			

Values are mean ± SD.

Δ and ΔΔ represent within‐group changes and between‐group differences in change from baseline, respectively, estimated from mixed models, with corresponding 95% confidence intervals. Global *P* values correspond to Type III tests of fixed effects from the mixed model.

Abbreviations: BDC, baseline period; C, control group; EX, exercise‐only group; EX‐AG, exercise plus artificial gravity group; HDT, head‐down tilt bed rest; RMR, resting metabolic rate; RQ, respiratory quotient.

**Table 6:** Indirect calorimetry comparison adjusted for FFM at HDT60 *vs*. baseline (BDC‐1)

Variable	Group	ΔHDT60	ΔΔHDT60	Global time *P*	Global group *P*	Global interaction *P*
**RMR (kcal 24 h^−1^)**	C	−102 (−203; −0.25)	EX *vs*. C: 29.3 (−97.4; 156); *P* = 0.628	0.024	0.922	0.054
	EX	−72.2 (−171; 26.6)	EX‐AG *vs*. C: 111 (8.35; 214); *P* = 0.037			
	EX‐AG	9.86 (−32.5; 52.2)	EX‐AG *vs*. EX: 82.1 (−18.8; 183); *P* = 0.099			
**RQ**	C	0.03 (0.01; 0.06)	EX *vs*. C: 0.00 (−0.03; 0.03); *P* = 0.980	0.001	0.113	0.100
	EX	0.03 (0.01; 0.06)	EX‐AG *vs*. C: −0.03 (−0.06; 0.00); *P* = 0.059			
	EX‐AG	0.00 (−0.02; 0.03)	EX‐AG *vs*. EX: −0.03 (−0.06; 0.00); *P* = 0.061			
**Carbohydrate utilization (mg min^−1^)**	C	20.9 (2.24; 39.5)	EX *vs*. C: 6.92 (−18.2; 32.0); *P* = 0.572	0.006	0.122	0.113
	EX	27.8 (9.34; 46.2)	EX‐AG *vs*. C: −19.8 (−45.8; 6.23); *P* = 0.128			
	EX‐AG	1.07 (−18.0; 20.1)	EX‐AG *vs*. EX: −26.7 (−52.7; −0.75); *P* = 0.044			
**Fat utilization (mg min^−1^)**	C	−19.7 (−31.2; −8.24)	EX *vs*. C: 0.24 (−15.6; 16.0); *P* = 0.975	<0.001	0.295	0.069
	EX	−19.5 (−30.9; −8.07)	EX‐AG *vs*. C: 17.0 (0.73; 33.3); *P* = 0.041			
	EX‐AG	−2.71 (−14.5; 9.13)	EX‐AG *vs*. EX: 16.8 (0.50; 33.0); *P* = 0.044			
**Protein utilization (mg min^−1^)**	C	9.71 (3.36; 16.0)	EX *vs*. C: −1.97 (−10.8; 6.85); *P* = 0.646	<0.001	0.92	0.730
	EX	7.73 (1.42; 14.1)	EX‐AG *vs*. C: −3.46 (−12.5; 5.60); *P* = 0.436			
	EX‐AG	6.25 (−0.30; 12.8)	EX‐AG *vs*. EX: −1.48 (−10.5; 7.56); *P* = 0.736			
**Urinary nitrogen excretion (g day^−1^**)	C	2.25 (0.77; 3.73)	EX *vs*. C: −0.46 (−2.52; 1.61); *P* = 0.651	<0.001	0.842	0.607
	EX	1.79 (0.32; 3.27)	EX‐AG *vs*. C: −1.00 (−3.07; 1.06); *P* = 0.324			
	EX‐AG	1.25 (−0.22; 2.71)	EX‐AG *vs*. EX: −0.55 (−2.61; 1.52); *P* = 0.588			

Δ and ΔΔ represent within‐group changes and between‐group differences in change from baseline, respectively, estimated from mixed models, with corresponding 95% confidence intervals.

Abbreviations: BDC, baseline period; C, control group; EX, exercise‐only group; EX‐AG, exercise plus artificial gravity group; HDT, head‐down tilt bed rest; RMR, resting metabolic rate; RQ, respiratory quotient.

Importantly, these between‐group differences remained detectable after adjustment for concurrent FFM (group‐by‐time interaction *P = *0.054), indicating that loss of lean mass did not fully account for the decline in RMR.

Substrate oxidation patterns derived from indirect calorimetry are presented in Table 5. During bed‐rest fat utilization declined markedly in C and EX (−22.1 and −21.6 mg min^−1^ respectively), whereas it was largely preserved in EX‐AG (−3.9 mg min^−1^). This resulted into greater preservation of fat oxidation in EX‐AG compared with both C and EX (ΔΔ = +18.2 and +17.7 mg min^−1^, *P* = 0.032 and 0.036, respectively), consistent with a differential group response over time (group‐by‐time interaction *P* = 0.054). Conversely, carbohydrate utilization increased in C and EX (+19.4 and +26.5 mg min^−1^, respectively), while remaining unchanged in EX‐AG (+0.3 mg min^−1^). Although the global group‐by‐time interaction was not statistically significant (*P* = 0.121), pairwise contrasts indicated lower carbohydrate reliance in EX‐AG compared with EX (ΔΔ = −26.2 mg min^−1^, *P* = 0.047).

RQ showed a similar pattern, increasing slightly in C and EX (+0.04), while remaining stable in EX‐AG (RQ = 0.81). When compared with C, EX‐AG showed a lower RQ change, in line with the better preservation of fat utilization, although the between‐group difference did not reach statistical significance (ΔΔ = −0.03, *P* = 0.051). These patterns were globally preserved after adjustment for FFM (Table 6).

### Exercise countermeasures fail to prevent bed rest‐induced reductions in muscle function

Changes in MCV between BDC‐4 and R+2 are summarized in Table 7. MVC torque decreased markedly across all groups in both knee extension (∼25%) and plantar flexion (∼20%). The magnitude of strength loss did not differ between groups, indicating that neither countermeasure effectively prevented the decline in maximal torque (all interaction and pairwise *P* > 0.20). Peak and 0.5 s averaged torque values showed consistent temporal patterns, with no meaningful differences between the two measures.

**Table 7:** Maximal voluntary isometric contraction (MVC) strength and countermovement jump (CMJ) performance values, and comparison at recovery *vs*. baseline

Variable	Group	BDC	R	ΔR	ΔΔR	Global time *P*	Global group *P*	Global interaction *P*
**Peak MVC – Ankle EXT (Nm)**	C	−133 (43)	−108 (30)	25.2 (10.1; 40.2)	EX *vs*. C: 11.4 (−9.92; 32.7); *P* = 0.279	<0.001	0.709	0.231
	EX	−148 (26)	−111 (26)	36.5 (21.5; 51.6)	EX‐AG *vs*. C: −6.58 (−27.9; 14.7); *P* = 0.528			
	EX‐AG	−129 (17)	−110 (15)	18.6 (3.52; 33.6)	EX‐AG *vs*. EX: −17.9 (−39.2; 3.35); *P* = 0.094			
**Peak MVC – Ankle FLEX (Nm)**	C	43.8 (8.5)	39.0 (6.6)	−4.78 (−7.62; −1.94)	EX *vs*. C: −1.44 (−5.45; 2.58); *P* = 0.466	<0.001	<.001	0.597
	EX	49.5 (5.6)	43.3 (6.4)	−6.22 (−9.06; −3.38)	EX‐AG *vs*. C: −1.91 (−5.93; 2.11); *P* = 0.334			
	EX‐AG	46.3 (3.8)	39.7 (4.3)	−6.69 (−9.53; −3.85)	EX‐AG *vs*. EX: −0.47 (−4.49; 3.54); *P* = 0.809			
**0.5 s MVC mean –Ankle EXT (Nm)**	C	−132 (43)	−104 (29)	27.6 (11.8; 43.5)	EX *vs*. C: 8.52 (−13.9; 30.9); *P* = 0.438	<0.001	0.673	0.274
	EX	−146 (26)	−110 (27)	36.2 (20.3; 52.0)	EX‐AG *vs*. C: −9.37 (−31.8; 13.0); *P* = 0.394			
	EX‐AG	−127 (18)	−108 (16)	18.3 (2.44; 34.1)	EX‐AG *vs*. EX: −17.9 (−40.3; 4.51); *P* = 0.112			
**0.5 s MVC mean – Ankle FLEX (Nm)**	C	43.2 (8.6)	38.3 (6.5)	−4.97 (−7.89; −2.05)	EX *vs*. C: −1.36 (−5.49; 2.77); *P* = 0.502	<0.001	0.252	0.535
	EX	48.6 (5.5)	42.3 (6.1)	−6.33 (−9.25; −3.41)	EX‐AG *vs*. C: −2.24 (−6.37; 1.89); *P* = 0.272			
	EX‐AG	45.6 (4.0)	38.3 (4.4)	−7.21 (−10.1; −4.29)	EX‐AG *vs*. EX: −0.88 (−5.01; 3.25); *P* = 0.661			
**Peak MVC – knee EXT (Nm)**	C	193 (39)	144 (27)	−48.3 (−67.3; −29.4)	EX *vs*. C: 2.78 (−23.1; 28.7); *P* = 0.825	<0.001	0.391	0.824
	EX	217 (44)	171 (41)	−45.6 (−63.3; −27.8)	EX‐AG *vs*. C: −4.66 (−30.6; 21.3); *P* = 0.712			
	EX‐AG	216 (44)	163 (35)	−53.0 (−70.7; −35.3)	EX‐AG *vs*. EX: −7.44 (−32.5; 17.6); *P* = 0.543			
**Peak MVC – knee FLEX (Nm)**	C	−99.6 (26.3)	−87.2 (31.0)	12.4 (−1.17; 25.9)	EX *vs*. C: 14.1 (−4.44; 32.7); *P* = 0.128	<0.001	0.449	0.257
	EX	−116 (16)	−89.9 (20.8)	26.5 (13.8; 39.1)	EX‐AG *vs*. C: 12.3 (−6.28; 30.8); *P* = 0.183			
	EX‐AG	−119 (17)	−94.3 (20.0)	24.6 (12.0; 37.3)	EX‐AG *vs*. EX: −1.85 (−19.8; 16.1); *P* = 0.832			
**0.5 s MVC mean – knee EXT (Nm)**	C	187 (41)	138 (24)	−48.9 (−68.5; −29.4)	EX *vs*. C: 3.02 (−23.7; 29.7); *P* = 0.816	<0.001	0.333	0.838
	EX	213 (43)	167 (42)	−45.9 (−64.2; −27.7)	EX‐AG *vs*. C: −4.33 (−31.1; 22.4); *P* = 0.739			
	EX‐AG	213 (45)	160 (35)	−53.3 (−71.5; −35.0)	EX‐AG *vs*. EX: −7.35 (−33.2; 18.5); *P* = 0.559			
**0.5 s MVC mean – knee FLEX (Nm)**	C	−97.0 (26.6)	−85.0 (29.9)	12.0 (−1.34; 25.3)	EX *vs*. C: 15.2 (−3.04; 33.4); *P* = 0.098	<0.001	0.44	0.204
	EX	−114 (17)	−87.4 (20.8)	27.1 (14.7; 39.6)	EX‐AG *vs*. C: 12.9 (−5.28; 31.2); *P* = 0.154			
	EX‐AG	−117 (17)	−91.9 (20.0)	24.9 (12.5; 37.3)	EX‐AG *vs*. EX: −2.24 (−19.8; 15.4); *P* = 0.793			
**Jumping height (m)**	C	0.31 (0.04)	0.24 (0.06)	−0.07 (−0.09; −0.05)	EX *vs*. C: 0.01 (−0.02; 0.05); *P* = 0.369	<0.001	0.131	0.65
	EX	0.35 (0.04)	0.29 (0.05)	−0.06 (−0.08; −0.03)	EX‐AG *vs*. C: 0.01 (−0.02; 0.04); *P* = 0.545			
	EX‐AG	0.31 (0.06)	0.26 (0.04)	−0.06 (−0.08; −0.04)	EX‐AG *vs*. EX: 0.00 (−0.04; 0.03); *P* = 0.764			
**Force (N)**	C	1467 (145)	1420 (279)	−50.6 (−152; 50.5)	EX *vs*. C: −120 (−263; 23.0); *P* = 0.095	0.002	0.846	0.205
	EX	1558 (167)	1370 (185)	−171 (−272; −69.5)	EX‐AG *vs*. C: −24.4 (−167; 119); *P* = 0.725			
	EX‐AG	1461 (152)	1371 (132)	−75.0 (−176; 26.1)	EX‐AG *vs*. EX: 95.6 (−47.4; 239); *P* = 0.178			
**Power (W)**	C	3018 (362)	2468 (458)	−589 (−737; −440)	EX *vs*. C: 16.3 (−194; 227); *P* = 0.872	<0.001	0.031	0.35
	EX	3499 (352)	2857 (338)	−572 (−721; −424)	EX‐AG *vs*. C: 137 (−73.6; 347); *P* = 0.189			
	EX‐AG	3037 (358)	2635 (326)	−452 (−601; −303)	EX‐AG *vs*. EX: 120 (−89.9; 331); *P* = 0.245			
**Velocity (m s^−1^)**	C	2.46 (0.17)	2.14 (0.30)	−0.31 (−0.42; −0.21)	EX *vs*. C: 0.09 (−0.05; 0.23); *P* = 0.212	<0.001	0.123	0.429
	EX	2.62 (0.16)	2.38 (0.20)	−0.23 (−0.33; −0.12)	EX‐AG *vs*. C: 0.06 (−0.08; 0.21); *P* = 0.367			
	EX‐AG	2.48 (0.22)	2.24 (0.18)	−0.25 (−0.35; −0.15)	EX‐AG *vs*. EX: −0.03 (−0.17; 0.12); *P* = 0.717			

Values are mean ± SD.

MVC assessments were performed at BDC‐4 and R+2, whereas jump tests were conducted at BDC‐3 and R+0. Δ and ΔΔ represent within‐group changes and between‐group differences in change from baseline, respectively, estimated from mixed models, with corresponding 95% confidence intervals. Global *P* values correspond to Type III tests of fixed effects from the mixed model.

Abbreviations: BDC, baseline period; C, control group; EX, exercise‐only group; EX‐AG, exercise plus artificial gravity group; EXT, extension; FLEX, flexion; MVC, maximal voluntary contraction; R, recovery period.

Explosive performance, assessed via CMJ at BDC‐2 and R+2, followed a similar pattern. Jump height decreased in all groups (C: −0.07 m; EX: −0.06 m; EX‐AG: −0.06 m, all *P* < 0.001), with no evidence of differential group responses over time (group‐by‐time interaction *P* = 0.65). Jump power, force and velocity also declined markedly over time (all *P* < 0.001), with none of the between‐group contrasts reaching statistical significance (all *P* > 0.10).

Overall, these findings indicate that muscle function was substantially reduced after prolonged bed rest, and that neither the exercise nor the combined exercise plus AG interventions were sufficient to preserve muscular performance.

### Exercise preserves maximal cycling power in a modality‐specific manner

Changes in maximal cycling power, derived from the incremental upright cardiopulmonary exercise tests performed at BDC‐2 and R+2, differed between groups (group‐by‐time interaction *P* < 0.001). Maximal power declined substantially in C (−59 W, *P* < 0.001), remained stable in EX (−6 W, *P* = 0.534) and increased in EX‐AG (+22 W, *P* = 0.038). Between‐group contrasts of changes from baseline confirmed greater preservation in both EX (*P* = 0.002), and in EX‐AG (*P* < 0.001) relative to C, whereas the difference between EX and EX‐AG changes was smaller (*P* = 0.057). Overall, these results indicate that the cycling‐based countermeasure was sufficient to preserve maximal power output, and that the addition of AG was associated with a net improvement, despite the absence of protection against muscle mass and maximal force loss. These findings are in line with previously reported changes in maximal oxygen uptake (Hedge et al., 2025).

### From RMR–FFM relationships to integrated multivariate models

#### Relationship between RMR and FFM

Changes in RMR (BDC‐1 to HDT60) were only moderately correlated with changes in FFM (BDC‐2 to HDT56), indicating that lean mass alone explained a limited proportion of the inter‐individual variability in resting metabolic adaptation (*R* ^2^ = 0.26, *P* = 0.010).

**Figure 1:**
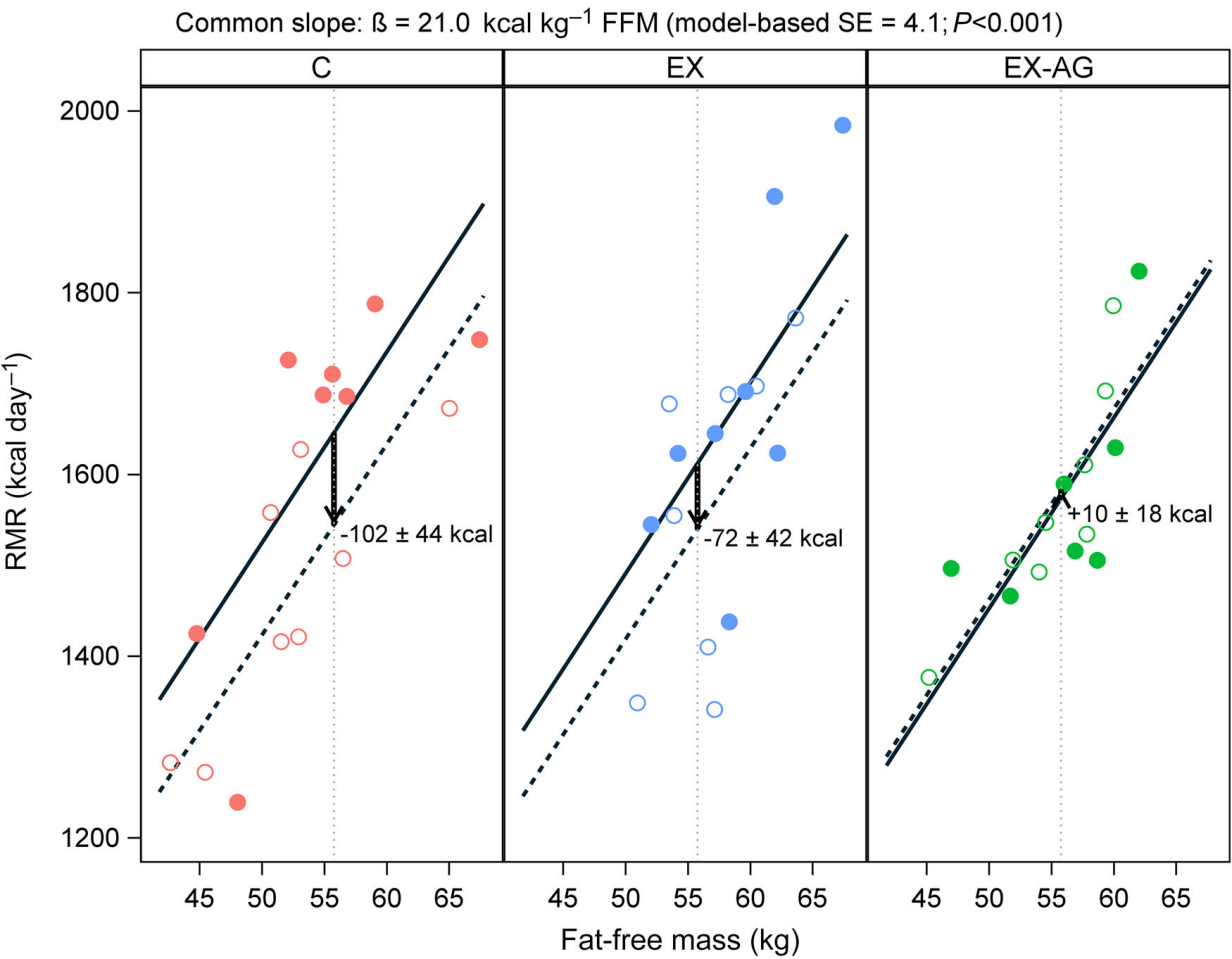
Model‐based relationship between resting metabolic rate (RMR) and fat‐free mass (FFM) at baseline (BDC) and after 60 days of head‐down tilt (HDT) Each panel displays the marginal relationship between RMR and FFM estimated from the linear mixed‐effects model for one intervention group (C, EX, EX‐AG). A common slope was estimated across groups (β = 21.0 kcal kg^−^^1^; model‐based SE = 4.1; *P* < 0.001). Filled circles represent individual baseline (BDC) measurements; open circles represent end‐HDT measurements. Solid and dashed lines denote the model‐estimated regression lines at BDC and HDT, respectively. Vertical arrows illustrate the model‐estimated shift in RMR at the mean FFM for each group (estimate ± model‐based SE). C, control group; EX, exercise‐only group; EX‐AG, exercise plus artificial gravity group. All participants were included in the analysis (*n* = 8 per group).

To further characterize this association, a longitudinal mixed‐effects model was fitted using repeated RMR and FFM measurements. As shown in Fig. 1, repeated RMR was still positively associated with repeated FFM (common slope β = 21.0 kcal kg^−^^1^; model‐based SE = 4.1; *P* < 0.001), indicating preserved scaling between lean tissue mass and resting energy expenditure across groups and time points. However, despite this stable slope, after 60 days of HDT, C and EX exhibited a downward shift in predicted RMR for a given FFM, whereas this shift was attenuated in EX‐AG (group‐by‐time interaction *P* = 0.054). At the mean FFM, the estimated shifts from baseline to HDT56 were −102 kcal in C, −72 kcal in EX and +10 kcal in EX‐AG. Compared with C, EX‐AG demonstrated a significantly smaller downward shift (+111 kcal; *P* = 0.037).

Overall, these results indicate that while FFM remains a key determinant of RMR, prolonged bed rest reduces RMR beyond what can be explained by lean tissue loss alone, and that the combination of exercise with AG attenuates this effect, preserving a higher RMR for a given FFM compared with control conditions.

#### Integrated analysis of RMR, muscle composition and performance

##### PCA of Δ‐variables

**Figure 2:**
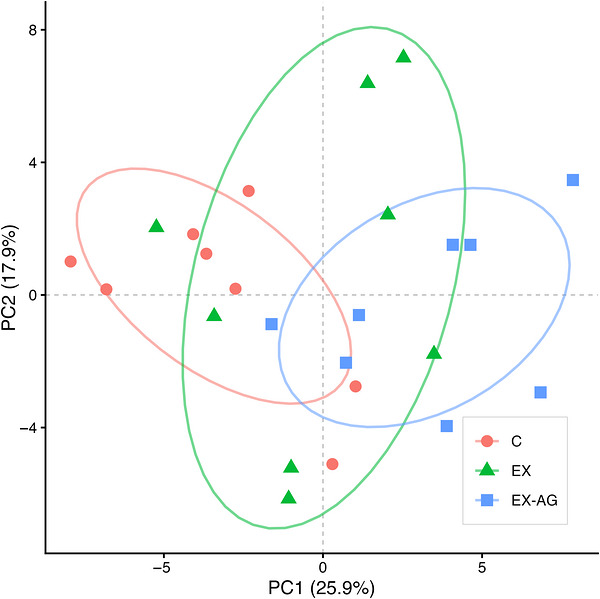
Principal components analysis score plot of Δ‐variables PC1 (25.9%) captured the primary axis of musculoskeletal–metabolic adaptation: EX‐AG participants clustered toward higher PC1 values (more favourable physiological changes), controls toward lower values (greater deterioration) and EX participants in between. PC2 (17.9%) represented additional, group‐independent variability. Ellipses represent 68% confidence regions (1 SD) for each group, illustrating within‐group variability and central tendency in the multivariate space. Abbreviations: PC, principal component; C, control group; EX, exercise‐only group; EX‐AG, exercise plus artificial gravity group. All participants were included in the analysis (*n* = 8 per group).

PCA performed on 62 Δ‐variables spanning MRI‐, DXA‐, performance‐ and metabolic‐derived indices revealed two main dimensions of coordinated physiological adaptation to bed rest and the countermeasures (Fig. 2). The first two principal components explained 43.8% of the total variance (PC1 = 25.9%; PC2 = 17.9%). PC1 represented a musculoskeletal preservation axis, dominated by coherent positive contributions from whole‐body and regional FFM indices: ΔTotal Thigh FFM Volume (4.75%), ΔTotal FFMI (MRI‐derived, 4.71%), ΔTrunk FFMI (4.71%), ΔLeft Anterior Thigh FFM Volume (MRI‐derived, 4.62%) and ΔTotal FFM (4.49%). Additional thigh‐specific indices (right anterior/posterior and left posterior MRI‐derived FFM volumes), lower‐body FFM/FFMI and trunk FFM contributed similarly (≈3–4%). These positive loadings were opposed by negative contributions from ΔFat Ratio (MRI‐derived in %) and ΔMass‐to‐Lean Mass Ratio (MRI‐derived), indicating that higher PC1 scores reflect superior preservation of skeletal muscle with relatively less fat accretion, whereas lower PC1 scores reflect more pronounced muscle loss and adverse body‐composition shifts. PC2, explaining 17.9% of the variance, described an adiposity‐related axis characterized by large positive contributions from ΔTotal Tissue Mass, ΔTotal Abdominal Adipose Tissue Index (MRI‐derived), ΔBM, ΔBMI and MRI‐derived abdominal fat depots (ΔASAT Volume, ΔVAT Volume and related indices; individual contributions ≈5%). Higher PC2 scores reflected coordinated gains in BM and central adiposity, while lower PC2 scores indicated stable or reduced fat stores.

Individual PCA scores showed clear group‐level structure: participants in EX‐AG tended to cluster toward higher PC1 values, consistent with better preservation of lean tissue and lower fat accumulation; control subjects clustered toward negative PC1 values, indicating greater muscle atrophy and fat gain; and EX exhibited intermediate responses. Together, these two orthogonal components summarize the main dimensions of integrated tissue‐level adaptation to HDT bed rest and provide a physiologically interpretable framework for the subsequent supervised sPLS analyses of ΔRMR.

##### sPLS analysis of ΔRMR

**Figure 3:**
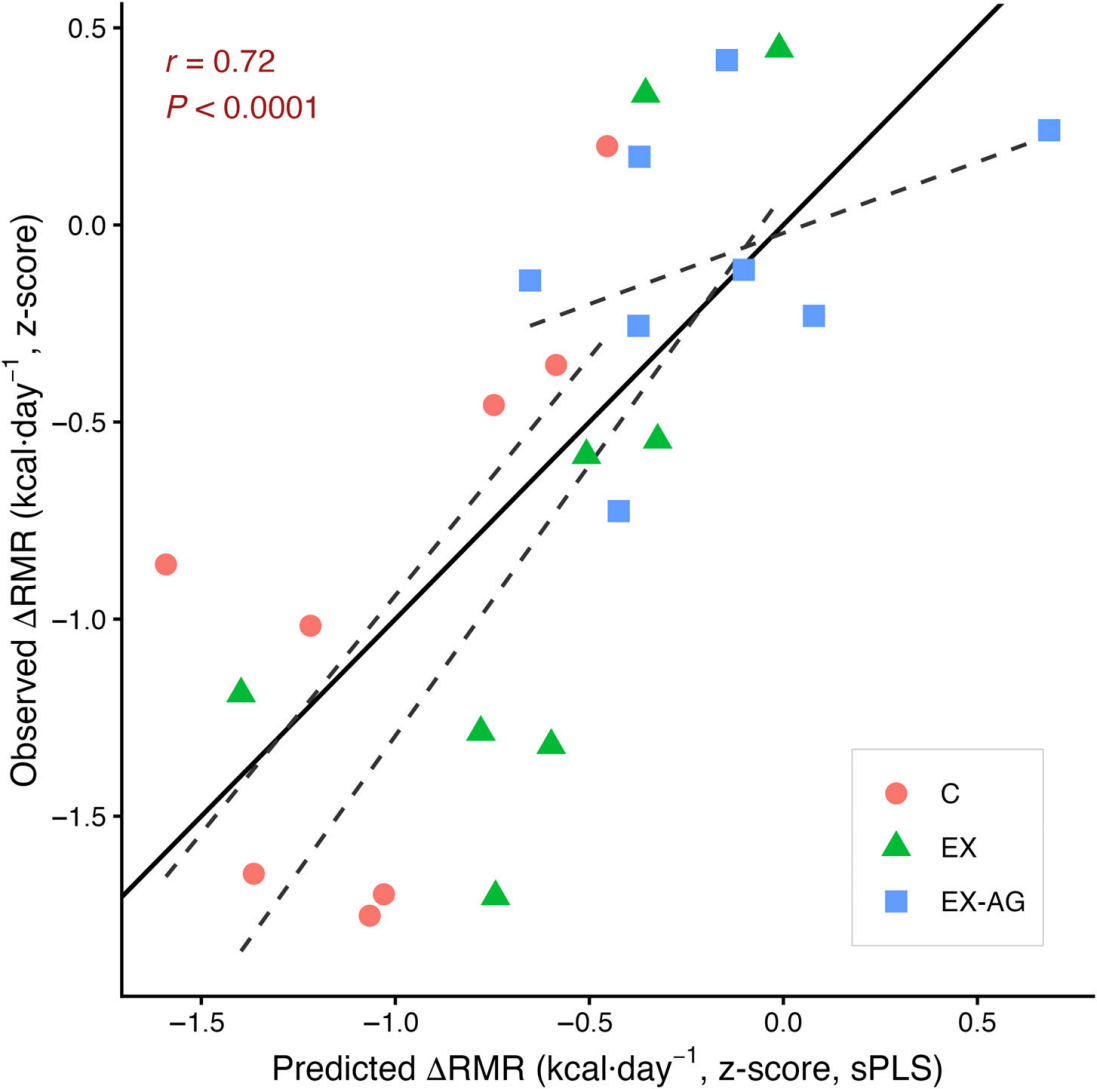
Agreement between observed and partial least squares‐predicted Δ resting metabolic rate values (one‐component model, keepX = 10) Individual predictions are shown for each participant, grouped by intervention. The identity line (solid) and regression fit (dashed) illustrate model performance (*r* = 0.72; *P* < 0.0001). Abbreviations: sPLS, partial least squares; RMR, resting metabolic rate; C, control group; EX, exercise‐only group; EX‐AG, exercise plus artificial gravity group. All participants were included in the analysis (*n* = 8 per group).

The sPLS analysis identified a coherent multivariate pattern associated with preservation of RMR during bed rest. This approach integrated information from the high‐dimensional set of 60 Δ‐variables relative to the sample size (*n* = 24). A single‐component sPLS model retaining 10 Δ‐variables (keepX = 10) showed a strong in‐sample association between observed and predicted ΔRMR (*r* = 0.72, *R* ^2^ = 0.52, *P* < 0.0001; Fig. 3). Although this reflects explanatory rather than predictive performance, it indicates that the retained latent axis captured a coherent multivariate pattern related to RMR preservation. Cross‐validation indicated modest predictive generalization (*R* ^2^ CV ≈ 0.12), supporting the interpretation of sPLS as a feature‐integration and hypothesis‐generating rather than predictive tool. The model retained 10 Δ‐variables with non‐zero loadings, indicating their contribution to the latent axis associated with ΔRMR. ΔRQ emerged as the dominant contributor to the latent axis, indicating that greater reliance on fat oxidation was strongly associated with preservation of RMR. Lower ΔRQ, reflecting a metabolic shift toward increased fat utilization, was associated with better preservation of RMR. The remaining selected predictors included Δ ${\dot{V}}_{O_{2}\mathrm{peak}}$ (mL kg^−^^1^ min^−^^1^), MRI‐derived thigh FFM volumes, DXA‐derived FFMI indices, urinary nitrogen excretion and knee flexion strength, jointly representing metabolic, morphological and neuromuscular adaptations associated with ΔRMR.

Taken together, the multivariate profile associated with higher preservation of RMR was characterized by: (1) a greater fat reliance (lower ΔRQ), (2) better maintenance of fat‐free tissues across trunk, upper‐body (arms + trunk) and thigh compartments, (3) preservation of aerobic capacity and (4) retention of knee flexor mechanical output. Conversely, participants exhibiting increases in RQ, reductions in lean compartments, and losses in cardiorespiratory or neuromuscular function showed greater declines in RMR. These findings highlight the integrated metabolic, morphological and functional adaptations supporting the maintenance of RMR during unloading and provide a physiologically interpretable multivariate framework to complement the exploratory PCA results.

## Discussion

The present study provides new insights into the metabolic and functional consequences of prolonged HDT bed rest and the partial efficacy of exercise‐based countermeasures. Cycling exercise combined with AG appeared to preserve RMR, whereas neither intervention prevented declines in maximal voluntary contraction or jump performance. These results suggest that metabolic and neuromuscular adaptations to bed rest may be partially dissociated, with AG primarily sustaining basal metabolic regulation while failing to maintain skeletal muscle contractile function.

In contrast to earlier bed rest investigations that primarily reported altered vascular control without marked increases in resting blood pressure (Aletti et al., 2012; Iwasaki et al., 2000), the present study revealed a distinct rise in DBP in the control group (+11.4 mmHg), while both countermeasures effectively blunted this response (EX +3.3 mmHg; EX‐AG +2.6 mmHg). These modest haemodynamic changes probably reflect the combined effects of cephalad fluid shift, altered baroreflex sensitivity and endothelial dysfunction under simulated microgravity (Baran et al., 2021). The maintenance of stable resting blood pressure in both countermeasures suggests that regular physical activity, even in the absence of AG, is sufficient to counteract the sympathetic and vascular remodelling typically observed during prolonged bed rest. It should be noted that these findings are based on resting haemodynamic measurements obtained under standardized conditions; cardiovascular responses during exercise were not systematically assessed and therefore cannot be inferred from the present data.

Beyond cardiovascular regulation, one of the most distinctive findings of this study is the preservation of RMR under combined EX‐AG. The maintenance of fasting energy expenditure in the EX‐AG group, despite moderate reductions in FFM, indicates that RMR preservation cannot be explained solely by structural tissue preservation. In line with the multivariate analyses, the variables most strongly associated with ΔRMR were indices of FFM and metabolic function rather than FM‐related metrics, indicating that changes in FM per se have limited influence on ΔRMR during bed rest.

A complementary mechanistic interpretation relates to substrate utilization. During bed rest, the control and EX groups shifted their fasting metabolism toward higher carbohydrate reliance, as reflected by higher RQ values while showing RMR declines. In contrast, EX‐AG participants maintained greater fasting fat oxidation and stable RQ, a pattern compatible with a higher internal energetic cost of exercise performed under AG. Although prescribed workloads were identical, exercise performed under AG may impose greater cardiovascular and mechanical demands due to orthostatic stress, reduced stroke volume, enhanced sympathetic activation, and additional stabilization or ventilatory work. These mechanisms are inferred from established physiological responses to head‐to‐foot gravitational loading, as cardiovascular responses were not systematically monitored during exercise in the present study. Despite similar high carbohydrate intake across groups, EX‐AG participants may therefore have entered fasting assessments with lower glycogen availability, favouring lipid oxidation. Because lipid oxidation is energetically more costly per unit of ATP than carbohydrate utilization, this may contribute to sustaining fasting energy expenditure and reflects enhanced metabolic flexibility between exercise and fast states.

FFM‐adjusted RMR decreased by approximately 100 kcal·24 h^−^^1^ in the control group, while remaining stable in EX‐AG, confirming a genuine metabolic preservation under combined exercise and AG. Consistent with these metabolic findings, previously published MRI‐derived results showed that the same EX‐AG intervention attenuated lower‐limb FFM loss by about 0.7 kg relative to controls, suggesting that gravitational loading may potentiate the anabolic metabolic effect of exercise under unloading conditions (Mandic et al., 2025). Previous investigations have shown that decreases in FFM only partly account for reductions in RMR during prolonged inactivity, implying a downregulation of cellular metabolic activity (Ferrando et al., 1996; Muller et al., 2013; Ritz et al., 1998), thought to reflect reduced mitochondrial activity and sympathetic drive. In this regard, the combined mechanical and cardiovascular loading induced by concurrent cycling and centrifugation (Clement et al., 2015; Evans et al., 2018; Fisher et al., 2025) may help sustain oxidative metabolism and substrate turnover by partially restoring physiological stimuli otherwise absent under (simulated) microgravity conditions.

In contrast, exercise alone induced only a partial attenuation of the decline in RMR. This finding aligns with previous bed rest and spaceflight analogue studies showing that aerobic or moderate‐intensity training without AG provides limited protection against metabolic downregulation (Bergouignan et al., 2011; Ferrando et al., 1996; Hargens & Vico, 2016; Ritz et al., 1998; Stein et al., 1999). The modest reduction in FFM‐adjusted RMR in the EX group (−72 kcal·24 h^−^^1^) may reflect the predominantly concentric and supine nature of training, which, although sufficient to maintain cardiorespiratory fitness (Hedge et al., 2025), might not provide sufficient mechanical or endocrine signals to sustain basal metabolic demand.

Beyond substrate selection, subtle differences in energy balance and adipose‐tissue dynamics may also have contributed to preserved lipid utilization and better maintenance of RMR in EX‐AG. Although none of the groups presented positive energy balance, EX‐AG participants showed small reductions in FM compared to the other groups, and almost no intramuscular fat infiltration (Mandic et al., 2025). In this context, such changes may reflect a greater reliance on lipid mobilization during fasting to meet energetic demands. Moreover, recent evidence indicates that nutrient oversupply during inactivity promotes intramyocellular lipid accumulation and metabolic inflexibility, thereby impairing mitochondrial function (Eggelbusch et al., 2024). Accordingly, the absence of energy surplus, together with maintained lipid utilization and small reductions in fat‐mass in EX‐AG, may have limited muscle lipid overload and preserved oxidative metabolism, thereby contributing to maintaining fasting energy expenditure under microgravity conditions.

Despite the metabolic preservation observed in EX‐AG, both exercise countermeasures failed to prevent the decline in neuromuscular function. MVC declined significantly and comparably across all groups (i.e. ≈25% in knee extension), with smaller but consistent reductions in plantar flexion strength (≈20%). MVC declined to a similar extent in all groups, indicating that neither exercise alone nor its combination with AG was sufficient to maintain maximal strength. Explosive performance followed a similar trajectory, with CMJ height significantly decreasing by ≈6–7 cm, and parallel reductions in jump power, force and velocity, again without differences between groups. These findings are consistent with previous long‐duration bed rest studies showing that aerobic training cannot fully preserve muscle force or power (Marusic et al., 2021). Functional decrements often exceed the degree of muscle atrophy, implicating impaired neural drive, excitation‐contraction coupling and tendon stiffness as key determinants of performance loss during unloading (Rudrappa et al., 2016). Together these results indicate that complete musculoskeletal preservation probably requires targeted high‐load resistive elements (Alkner & Tesch, 2004; Trappe et al., 2007). In contrast, the exercise countermeasures were successful in maintaining maximal power output, which is in line with previous results regarding ${\dot{V}}_{O_{2}\mathrm{peak}}$ (Hedge et al., 2025). Overall, this indicates that cycling and cycling plus AG can be used to maintain endurance capacity during long‐term microgravity (simulation).

The results from the present study should be interpreted with some considerations in mind. The relatively small sample size (*n* = 8 per group) inevitably limits statistical power and increases uncertainty around effect estimates, particularly those of smaller amplitude. Accordingly, these findings should be interpreted with caution, as limited power may reduce sensitivity to detect subtle yet physiologically meaningful differences. In this context, although the sPLS model showed moderate explanatory capacity, its predictive performance under cross‐validation was modest; therefore, the multivariate associations should be viewed as hypothesis‐generating rather than predictive. In addition, although protein intake and its distribution were standardized across groups, these factors remain important modulators of muscle protein turnover and metabolic adaptations during bed rest and may partly explain inter‐individual variability. Another aspect to consider for future studies using cycling exercise as a countermeasure is to adjust the cycling loads during the intervention to match potential adaptations and thus optimize the training stimulus. Finally, the exclusive inclusion of male participants limits the generalizability of the present findings. Given that female astronauts may be exposed to distinct endocrine and metabolic environments during space missions, further investigation is warranted to determine whether sex‐specific physiological or hormonal factors modulate the response to (simulated) microgravity and countermeasures employing AG.

### Conclusions

Taken together, the present findings indicate that AG, when combined with aerobic exercise, may help preserve metabolic stability during prolonged bed rest, yet its influence on neuromuscular integrity remains limited. This partial efficacy reinforces the notion that distinct physiological systems respond differentially to (simulated) microgravity and to countermeasures aimed at mitigating it. Beyond its relevance to spaceflight, such dissociation between metabolic and mechanical adaptations provides valuable insight into the regulation of energy expenditure and muscle function in other contexts of severe inactivity, such as immobilization or ageing. Future investigations should therefore prioritize integrated countermeasure models that combine gravitational, metabolic and neuromuscular stimuli to achieve a more comprehensive protection against the multifaceted deconditioning induced by microgravity.

## Additional information

## Competing interests

The authors declare no competing interests relevant to the content of this paper.

## Author contributions

C.L., M.P.B., R.B.d.V., R.F.G., C.S.: conception and design of the work. S.M.R., M.M., C.L., R.B.d.V., R.F.G., C.S.: acquisition, analysis and interpretation of data for the work. S.M.R., R.F.G., C.S.: drafting of the manuscript. S.M.R., M.M., C.L., M.P.B., R.B.d.V., R.F.G., C.S.: revising the manuscript critically for important intellectual content. All authors approved the final version of the manuscript and agree to be accountable for all aspects of the work in ensuring that questions related to the accuracy or integrity of any part of the work are appropriately investigated and resolved. All persons designated as authors qualify for authorship, and all those who qualify for authorship are listed.

## Funding

The BRACE study was funded by ESA and CNES. CNES was the Promoter of the study according to French law. The work presented here was further supported by grants from the Swedish National Space Agency (SNSA) to R.F.G. (#2020‐00141 and #2023‐00205) and from the CNRS to C.L. (#2023/4800001217 and 2024/41334/00). R.F.G. is supported by a career grant from the Swedish National Space Agency (#2021‐00159).

## Supporting information


Peer Review History

## Data Availability

All data supporting the findings of this study are available from the corresponding author on request.
