# Supplementary material for: Artificial gravity during prolonged bed rest preserves resting metabolic rate but not muscle function: Evidence from the BRACE study
Source: J Physiol. 2026 May 24;604(12):4771–94. doi: 10.1113/JP290788 (PMC13267683; doi:10.1113/JP290788)
Supplement: Supplementary file 1 — Peer Review History [file TJP-604-4771-s001.pdf]

The Journal of Physiology

Artificial Gravity During Prolonged Bed Rest Preserves Resting Metabolic Rate but not Muscle Function: Evidence from the BRACE Study

Saul Martin Rodriguez, Mirko Mandić, Claire Laurens, Marie-Pierre Bareille, Rebecca Billette de Villemeur, Rodrigo Fernandez-Gonzalo, and Chantal Simon
DOI: 10.1113/JP290788

Corresponding author(s): Rodrigo Fernandez-Gonzalo (rodrigo.fernandez-gonzalo@ki.se)

The referees have opted to remain anonymous.

Review Timeline:

Submission Date:	19-Dec-2025
Editorial Decision:	17-Feb-2026
Revision Received:	02-Mar-2026
Editorial Decision:	10-Apr-2026
Revision Received:	16-Apr-2026
Accepted:	22-Apr-2026

Senior Editor: Karyn Hamilton

Reviewing Editor: Ross Pollock

Transaction Report:

(Note: With the exception of the correction of typographical or spelling errors that could be a source of ambiguity, letters and reports are not edited. Depending on transfer agreements, referee reports obtained elsewhere may or may not be included in this compilation. Referee reports are anonymous unless the Referee chooses to sign their reports.)

Re: JP-RP-2025-290788 "**Artificial Gravity During Prolonged Bed Rest Preserves Resting Metabolic Rate but not Muscle Function: Evidence from the BRACE Study**" by Saul Martin Rodriguez, Mirko Mandić, Claire Laurens, Marie-Pierre Bareille, Rebecca Billette de Villemeur, Rodrigo Fernandez-Gonzalo, and Chantal Simon

Dear Dr Fernandez-Gonzalo,

Thank you for submitting your manuscript to The Journal of Physiology. It has been assessed by a Reviewing Editor and by 2 expert referees and we are pleased to tell you that it is potentially acceptable for publication following satisfactory major revision.

Please advise your co-authors of this decision as soon as possible.

The referee reports are copied at the end of this email.

Please address all the points raised and incorporate all requested revisions or explain in your Response to Referees why a change has not been made. We hope you will find the comments helpful and that you will be able to return your revised manuscript within 2 months. If your article is NOT for a Special Issue, you may have 9 months to revise. If you require an extension, please contact journal staff: jp@physoc.org. Please note that this letter does not constitute a guarantee for acceptance of your revised manuscript.

Your revised manuscript should be submitted online using the link in your Author Tasks: Link Not Available. This link is accessible via your account as Corresponding Author; it is not available to your co-authors. If this presents a problem, please contact journal staff (jp@physoc.org). Image files from the previous version are retained on the system. Please ensure you replace or remove any files that are being revised.

If you do not wish to submit a revised version of your manuscript, you must inform our journal staff (jp@physoc.org) or reply to this email to request withdrawal. Please note that a manuscript must be formally withdrawn from the peer review process at one journal before it may be submitted to another journal.

TRANSPARENT PEER REVIEW POLICY: To improve the transparency of its peer review process, The Journal of Physiology publishes online as supporting information the peer review history of all articles accepted for publication. Readers will have access to decision letters, including Editors' comments and referee reports, for each version of the manuscript, as well as any author responses to peer review comments. Referees can decide whether or not they wish to be named on the peer review history document.

ABSTRACT FIGURES: Authors are expected to use The Journal's premium BioRender account to create/redraw their Abstract Figures. Information on how to access this account is here: https://physoc.onlinelibrary.wiley.com/journal/14697793/biorender-access.

This will enable Authors to create and download high-resolution figures. If authors have used the free BioRender service, they can use the instructions provided in the link above to download a high-resolution version suitable for publication.

The link provided should only be used for the purposes of this submission. Authors will be charged for figures created on this account if they are not related to this manuscript submission.

LANGUAGE EDITING AND SUPPORT FOR PUBLICATION: If you would like help with English language editing, or other article preparation support, Wiley Editing Services offers expert help, including English Language Editing, as well as translation, manuscript formatting, and figure formatting at www.wileyauthors.com/eeo/preparation. You can also find resources for Preparing Your Article for general guidance about writing and preparing your manuscript at www.wileyauthors.com/eeo/prepresources.

RIGOUR AND REPRODUCIBILITY: We recommend authors consult The Journal's Rigour and Reproducibility requirements to ensure that their manuscript is suitable for publication: https://physoc.onlinelibrary.wiley.com/pb-assets/hub-assets/physoc/documents/TJP-Rigour-and-Reproducibility-Requirements.pdf

REVISION CHECKLIST:

Check that your Methods section conforms to journal policy: https://jp.msubmit.net/cgi-bin/main.plex?form_type=display_requirements#methods.

Check that data presented conforms to the statistics policy: https://jp.msubmit.net/cgi-bin/main.plex?form_type=display_requirements#statistics.

Upload a full Response to Referees file. To create your 'Response to Referees': copy all the reports, including any comments from the Senior and Reviewing Editors, into a Microsoft Word, or similar, file and respond to each point, using font or background colour to distinguish comments and responses and upload as the required file type.

Please upload two versions of your manuscript text: one with all relevant changes highlighted and one clean version with no changes tracked. The manuscript file should include all tables and figure legends, but each figure/graph should be uploaded as separate, high-resolution files.

You may also upload:

- 'Potential Cover Art' for consideration as the issue's cover image
- Appropriate Supporting Information (Video, audio or data set: see https://jp.msubmit.net/cgi-bin/main.plex?form_type=display_requirements#supp).

We look forward to receiving your revised submission.

If you have any queries, please reply to this email and we will be pleased to advise.

Yours sincerely,

Karyn Hamilton
Senior Editor
The Journal of Physiology

REQUIRED ITEMS

- Author photo and profile. First or joint first authors are asked to provide a short biography (no more than 100 words for one author or 150 words in total for joint first authors) and a portrait photograph. These should be uploaded and clearly labelled together in a Word document with the revised version of the manuscript. See Information for Authors for further details.

- You must start the Methods section with a paragraph headed Ethical Approval. If experiments were conducted on humans, confirmation that informed consent was obtained, preferably in writing, that the studies conformed to the standards set by the latest revision of the Declaration of Helsinki and that the procedures were approved by a properly constituted ethics committee, which should be named, must be included in the article file. If the research study was registered (clause 35 of the Declaration of Helsinki), the registration database should be indicated, otherwise the lack of registration should be noted as an exception (e.g. The study conformed to the standards set by the Declaration of Helsinki, except for registration in a database). For further information see: https://physoc.onlinelibrary.wiley.com/hub/human-experiments.

- The reference list must be in alphabetical order, rather than numbered, to comply with our Journal format.

- Your manuscript must include a complete Additional Information section, including competing interests; funding; author contributions and acknowledgements.

- Your paper contains Supporting Information of a type that we no longer publish, including supplementary tables and figures. Any information essential to an understanding of the paper must be included as part of the main manuscript and figures. The only Supporting Information that we publish are video and audio, 3D structures, program codes and large data files. Your revised paper will be returned to you if it does not adhere to our Supporting Information Guidelines.

- Papers must comply with the Statistics Policy: https://jp.msubmit.net/cgi-bin/main.plex?form_type=display_requirements#statistics.

In summary:

- If n {less than or equal to} 30, all data points must be plotted in the figure in a way that reveals their range and distribution. A bar graph with data points overlaid, a box and whisker plot or a violin plot (preferably with data points included) are acceptable formats.

- If n > 30, then the entire raw dataset must be made available either as supporting information, or hosted on a not-for-profit repository, e.g. FigShare, with access details provided in the manuscript.

- 'n' clearly defined (e.g. x cells from y slices in z animals) in the Methods. Authors should be mindful of pseudoreplication.

- All relevant 'n' values must be clearly stated in the main text, figures and tables.

- The most appropriate summary statistic (e.g. mean or median and standard deviation) must be used. Standard Error of the Mean (SEM) alone is not permitted.

- Exact p values must be stated. Authors must not use 'greater than' or 'less than'. Exact p values must be stated to three significant figures even when 'no statistical significance' is claimed.

EDITOR COMMENTS

Reviewing Editor:

Thank you for submitting your manuscript to the Journal of Physiology. It has been reviewed by two expert reviewers. In the review it is highlighted that greater clarity around the description of the exercise performed and also determination of energy intake. It was also noted that in places there was a statistical narrative which could be improved by having a more physiological narrative. Please also ensure the comments relating to the tables are also considered to improve clarity.

Please also see 'Required Items' above.

Senior Editor:

Comments for Authors to ensure the paper complies with the Statistics Policy (Required):
Please revisit The Journal's statistics policy to ensure that your revised manuscript is in full compliance. For example, some references to variability represented as SE appears in the figure legends, but the statistics section indicates that SD is used (The Journal requires SD rather than SE).

Comments to the Author:
Thank you for submitting your manuscript for consideration by The Journal of Physiology. As part of the peer review process, we recruited two Referees with expertise in this field of study. While both Referees note that your manuscript has the potential to be influential on this field of study, they also both raise major concerns that need to be satisfactorily addressed for the impact to be realized. At this stage, we would like to invite you to respond point-by-point to each Referee's concerns, making corresponding revisions to your manuscript. Please also bring the revised paper into compliance with The Journal's statistics policy and indicate whether or not the studies conformed to the standards set by the latest version of the Declaration of Helsinki (or the version that was in place at the time of the experiments). As there are no restrictions on page length, The Journal does not usually accept supplemental/supporting material that cannot be included with the body of the manuscript. Please read The Journal's policy regarding Supporting Information and contact The Journal staff with questions about your supplementary tables. We look forward to seeing your revised work and appreciate your interest in The Journal of Physiology.

REFEREE COMMENTS

Referee #1:

This is an interesting manuscript on the use of artificial gravity to counteract metabolic and muscle deconditioning due to long-term bed rest. The use of artificial gravity as a countermeasure is a very promising and timely intervention, in this respect the manuscript is original.

The results obtained show that cycling plus artificial gravity (EX-AG) is effetive in preserving resting metabolic rate but not in preventing neuromuscular decline measured in terms loss of maximum isometric force and peak jumping power of the lower limbs. However, both AG and EX-AG seemed effective in reducing the loss of fat free mass evaluated by DXA.

In this respect AG seems promising, while the addition of EX based on high-intesity cycling bouts does not seem to afford additional benefits, at least in the present form.

In this respect, it is difficult to understand the precise protocol used by the Authors for the cycling intervention; it seems they used a form of high-intensity interval training exercise (HIIT) but the precise details ahve been omitted. This raises the doubt

that the intensity of the HIIT protocol used was insufficient to protect against muscular deconditioning. Since training was based on high-intesity cycling, why was not maximum cycling power measured before and after bed rest? This would have been more specific than measuring isometric MVC or jumping power and could have shown differences.

One negative remark about the paper is that it is mostly written using a statistics-based narrative rather that a physiological-based narrative and as such it is hard to read.

Referee #2:

General comments

The manuscript reports on the efficacy of cycle ergometry as a spaceflight countermeasure. In addition, the study investigated whether the outcome of a daily cycle ergometry exercise would be improved, were the exercise conducted during artificial gravity (AG). AG was established with a short arm human centrifuge. This study was part of a larger study supported by the European Space Agency: Bedrest, Artificial gravity and Cycle Exercise, or BRACE for short.

Subjects were confined to 6{degree sign}head down bed rest for 60 days, and were separated into 3 groups: a control group, a group that conducted the cycle ergometry exercise in the horizontal position, and a group that conducted the cycle ergometry exercise in the horizontal position,while being exposed to a head-to-foot gravitational vector during centrifugation. The results indicate that both exercise countermeasures were not capable of mitigating the loss of muscle mass and strength, but preserved metabolic stability.

The article is well written, the results clear and the conclusions are appropriate.

Impact on the area of research

Comsidering the new era of space travel, both explorative (missions to the Moon and Mars) and touristic (low Earth orbit) in nature, the science of human spaceflight must match the engineering developments. An optimal countermeasure has not yet been developed, that would prevent adaptation of physiological systems to weightlessness and/or reduced gravity. The development of such countermeasures is a slow and arduous process. This manuscript evaluates a new strategy , that of artificial gravity combined wuth cycle ergoemtry. As a consequence, the report is extremely important in the field of exercise countermeasures, but not not only for space travel, but also for populations on Earth that exhibit muscle loss (i.e., age related muscle atrophy).

The research is highly original. To my knowledge, no long/term studies have been conducted on healthy young inactive individuals to assess the benefit of artifical gravity during cycle ergometry.

Major comments

1. The determination of the energy intake of the individual participants and the three groups is not clear. The section describing the determination of energy intake is confusing.

2. The prescription of the exercise is vague. This study evaluates a training programme comprised of cyce ergometry. Presumablty participants received personalised training programmes. How was this determined?

3. Participants in the artifical gravity groups were most likely experiencing some effects of the centrifugation (i.e., motion sickness). Did this affect their training programme compared to the EX group? Was the training the same for both groups? Any reports of muscle soreness that may have impacted on the ability of the participants to conduct the daily exercise? Did the motion sickness affect their appetite?

Minor comments

Line 116: This is essentially a study evaluating an exercise training programme. As such, it would benefit the reader to have some more detailed information regarding the exercise protocol. For example, what is meant by "standardised physical activity"? If the subjects were confined, how could they be very active?

Line 117: By "countermeasure" you mean exercise training. Although perhaps redundant for the space life scientists, it might be helpful to define this for others.

Line 129: What was the "standardised rehabilitation programme"? The term "standardised" is used throughout the text. Does this imply "personalised"? Was the the physical activity (see above) before the bedrest the same for all subjects? Was the exercise training intensity the same for all subjects?

Line 138 - 141: Presumably in such a confinement study incorporating an exercise training protocol, the energy balance is of utmost importance. What food data bases were used to determine the enrgy intake? The micronutrients were cotnrolled, as explained later in the text, what about macronutrients? What was the difference in the caloric intake between the groups? was there a large individual difference betwen subjects?

Line 142: Presumably three of the four meals were breakfast, lunch and dinner, what was the fourth? Afternoon or morning snack? How was the exercise scheduled between these meaals?

Line 143 - 155: This section is rather confusing. It is stated in the section prior to this that the participants received meals "tailored to match the energy requirements". It is also stated that the energy intake was between 90% and 110% of the energy requirement for 40 of the 60 days of confinement. How was the energy intake determined for each participant? Did this apply to all three groups or just the exercising group? In the event that the diet was hypocaloric, would this contribute to the loss of muscle mass, despite daly exercise?

Line 151: You state that "early monitoring of body mass and composition" suggested that the energy requirements were underestimated. In the previous paragraoh it is stated that the energy intakes were modified due to the requirements of a "scientific team"? Firstly, it might be helpful to explain that other teams were involved in the study. Secondly, this section requires much more detail. Body mass and composition is a function of the daily activity and energy intake (quality and quantity). From the description provided, it would appear that only the exercise was controlled. There is no disussion provided on how this variability in the energy intake affected body mass and composition.

Perhaps a timeline would help in understanding this. Participants were provided with an energy intake of 130% of resting metabolic rate during HDT 1 to HDT 11. They were then on a new schedule from HDT 11 to HDT 51, whereby the energy intake was between 90 and 110% of estimated requirements. Whan was the energy intake increased to 142%?

Line 156: Was it 1 or 1.2? Did it differ for the participants? Groups?

Line 159: What about the macronutrient recommendations?

Line 171: You use the term orthostasis during cenrtifugation. Normally, orthostasis refers to the physiological responses to standing upright, whereas g-tolerance is the response to elevated head to foot gravitational vector. Is OT the correct term here? Orthostatic tolerance is normally assessed by tilting the participant to 80{degree sign} upright and applying a reduced

pressure around the lower body (lower body reduced/negative pressure). I am not sure how this measurement is critical for your results?

Line 181: Was the exercise conducted as a percentage of the upright or supine VO2max? Please define. With time the daily training most likely modified the relative workload compatedf to the absolute workload. In other words, 40% of VO2max at the start of the bed rest may have change by the end of the bed rest. In the event that there was a positive effect, then this may be reduced (i.e., to 30% VO2max), or increased as a result of a negative effect. Was the VO2max measured at the end of the bed rest? Was the exercise intensity modified during the bed rest, as it would be during a treaining programme?

Line 236: The Quark device has an alcohol burner for calirating the RMR dertermined with the canopy. Was the gas calibration conducted in addition to the alcohol calibration? Could you be more specific as to how the gas calibration was conducted? Was this conducted with the canopy positioned on a surface (i.e., pillow)? The Quark system draws the gas from the canopy at a predetermined rate. By introducing an additional flow of gas into the canopy are you not affecting the measurement of flow?

Line 258: Was familiarisation conducted before the actual session (on another day) or just prior to the measurement?

Line 365: Since blood pressure was measured at rest, the measurement is not a "response" to a stimulus. Was the blood pressure response to the exericse affected? It would be helpful, if you reported the heart rate and blood pressure during the exercise over the 60-d period.

Line 546: How would the varying energy intake affect RMR and substrate utilisation?

Line 557: The authors state that "artificial gravity likely increased cardiovascular....demands". Were the cardiovascular responses not monitored during the exercise?

Table 1: The title of the Table suggests that the DXA values are compared by group at R+3 and BDC-1. The BDC column should then read BDC-1. The Table has two additional columns HDT60 and ΔHDT60. Should the title of the Table be modified to reflec this? Where was the moving average applied? Would it not be better to indicate that the Table represents values releavitve to BDC-1, rather than a "comparison"?

Table 2: Indriect calorimetry values at HDT60 relative to baseline?

Table 3: I am no tsure I understand this Table. The title suggests that it presents indirect calorimetry values adjusted for FFM at HDT60 relative to baseline. There are no indirect calorimetry values in this Table, only DXA values? Also, there are no values for HDT60, but for HDT10, HDT44, HDT56 the BDC colimn should probably be defined as BDC-1). If these values are relative to BDC, why have a BDC column?

END OF COMMENTS

EDITOR COMMENTS

Reviewing Editor:

Thank you for submitting your manuscript to the Journal of Physiology. It has been reviewed by two expert reviewers. In the review it is highlighted that greater clarity around the description of the exercise performed and also determination of energy intake. It was also noted that in places there was a statistical narrative which could be improved by having a more physiological narrative. Please also ensure the comments relating to the tables are also considered to improve clarity.

Please also see 'Required Items' above.

Senior Editor:

Comments for Authors to ensure the paper complies with the Statistics Policy (Required):

Please revisit The Journal's statistics policy to ensure that your revised manuscript is in full compliance. For example, some references to variability represented as SE appears in the figure legends, but the statistics section indicates that SD is used (The Journal requires SD rather than SE).

Comments to the Author:

Thank you for submitting your manuscript for consideration by The Journal of Physiology. As part of the peer review process, we recruited two Referees with expertise in this field of study. While both Referees note that your manuscript has the potential to be influential on this field of study, they also both raise major concerns that need to be satisfactorily addressed for the impact to be realized. At this stage, we would like to invite you to respond point-by-point to each Referee's concerns, making corresponding revisions to your manuscript. Please also bring the revised paper into compliance with The Journal's statistics policy and indicate whether or not the studies conformed to the standards set by the latest version of the Declaration of Helsinki (or the version that was in place at the time of the experiments). As there are no restrictions on page length, The Journal does not usually accept supplemental/supporting material that cannot be included with the body of the manuscript. Please read The Journal's policy regarding Supporting Information and contact The Journal staff with questions about your supplementary tables. We look forward to seeing your revised work and appreciate your interest in The Journal of Physiology.

Authors' response (AR): We thank the Reviewing and Senior Editors for their careful assessment of our manuscript and for the constructive guidance provided. We appreciate the recognition of the manuscript's potential impact, as well as the clear identification of the key areas requiring improvement.

In the revised version, we have substantially expanded and clarified the description of the exercise intervention and the determination of energy intake, in direct response to the Editors' comments and those of the Referees. We have also revised the Results and Discussion to improve the physiological narrative and reduce the emphasis on purely statistical reporting, where appropriate, thereby enhancing the interpretability of the findings.

We have carefully reviewed all tables and figures to improve clarity and internal consistency, and we have corrected issues related to table titles, time-point definitions, and baseline references. In addition, the manuscript has been fully revised to comply with *The Journal of Physiology* statistics policy, including consistent use of standard deviation (SD) for descriptive data and appropriate reporting of model-derived estimates.

The Methods section has been updated to explicitly state ethical approval and compliance with the Declaration of Helsinki, and all required items requested by the Journal have been addressed. Supporting material has been revised in accordance with the Journal's policy.

We believe that these revisions have substantially strengthened the manuscript and addressed all concerns raised by the Editors and Referees. We thank the Editors again for the opportunity to revise our work and for their valuable input.

REFEREE COMMENTS

Referee #1:

This is an interesting manuscript on the use of artificial gravity to counteract metabolic and muscle deconditioning due to long-term bed rest. The use of artificial gravity as a countermeasure is a very promising and timely intervention, in this respect the manuscript is original.

The results obtained show that cycling plus artificial gravity (EX-AG) is effective in preserving resting metabolic rate but not in preventing neuromuscular decline measured in terms loss of maximum isometric force and peak jumping power of the lower limbs. However, both AG and EX-AG seemed effective in reducing the loss of fat free mass evaluated by DXA.

In this respect AG seems promising, while the addition of EX based on high-intensity cycling bouts does not seem to afford additional benefits, at least in the present form.

AR: We thank Referee #1 for their thoughtful and constructive evaluation of our manuscript. We greatly appreciate their positive assessment of the originality and timeliness of the study, as well as their recognition of the potential of artificial gravity as a countermeasure to metabolic and musculoskeletal deconditioning. We are also grateful for their careful interpretation of our results and for the insightful comments regarding the exercise protocol, outcome specificity, and overall narrative structure of the manuscript. These comments have been very helpful in improving the

clarity of the Methods, refining the physiological interpretation of the findings, and strengthening the overall presentation of the revised manuscript. All points raised are addressed in detail below.

In this respect, it is difficult to understand the precise protocol used by the Authors for the cycling intervention; it seems they used a form of high-intensity interval training exercise (HIIT), but the precise details have been omitted. This raises the doubt that the intensity of the HIIT protocol used was insufficient to protect against muscular deconditioning. Since training was based on high-intensity cycling, why was not maximum cycling power measured before and after bed rest? This would have been more specific than measuring isometric MVC or jumping power and could have shown differences.

AR: We thank the Reviewer for this pertinent and constructive comment. We agree that, for an intervention based on high-intensity cycle ergometry, maximal cycling power constitutes a modality-specific outcome that complements neuromuscular measures such as MVC and jumping performance.

Maximal cycling power was in fact assessed during the incremental supine cardiopulmonary exercise test used to determine $\dot{V}O_2$peak. This test was performed during the baseline data collection phase (BDC-2) and repeated after the 60-day head-down tilt bed rest period during early recovery (R+2), in accordance with the BRACE protocol. These data were not included in the original submission and have now been added to the Results section of the revised manuscript.

From a physiological perspective, maximal cycling power provides an integrated index of central and peripheral adaptations, reflecting cardiovascular capacity, neuromuscular coordination, and the ability to generate high external power in the trained modality. Analysis using a mixed-effects model revealed a significant group-by-time interaction for maximal cycling power ($p < 0.001$). Maximal cycling power declined markedly in the control group (CTRL; −59 W, $p < 0.001$), remained essentially unchanged in the exercise-only group (EX; −6 W, $p = 0.534$), and increased in the exercise plus artificial gravity group (EX-AG; +22 W, $p = 0.038$). Between-group contrasts of changes from baseline demonstrated significant preservation in both EX ($p = 0.002$) and EX-AG ($p < 0.001$) relative to C, whereas the difference between EX and EX-AG was smaller ($p = 0.057$).

Overall, these findings indicate that the cycling-based countermeasure effectively preserved maximal cycling performance, and that the addition of artificial gravity provided an additional physiological stimulus sufficient to elicit improvements in this task-specific outcome. Importantly, the dissociation between preserved or improved cycling power and the absence of protection against muscle mass and maximal force loss highlights the mode-specific nature of training adaptations under bed rest conditions. Together, these results strengthen the interpretation of the intervention by demonstrating that, despite limited efficacy for

neuromuscular endpoints, the applied exercise stimulus was physiologically meaningful and effective within the trained modality.

One negative remark about the paper is that it is mostly written using a statistics-based narrative rather that a physiological-based narrative and as such it is hard to read.

AR: We thank the Reviewer for this thoughtful comment regarding the narrative style of the manuscript. We agree that clarity and readability are essential, particularly in a study that integrates complex physiological and statistical analyses. While we believe that the statistical information presented in the Results section is necessary and appropriate to support the findings, we acknowledge that in some instances the ordering of information may have foregrounded the statistical outcomes before the physiological interpretation.

To address this concern, we have revised several sentences in the Results section to place greater emphasis on the physiological observations first, followed by the corresponding statistical support. These changes do not alter the content or conclusions of the analyses but aim to improve the flow and accessibility of the Results for the reader. Minor adjustments were also made in the Discussion to further enhance narrative continuity.

We hope that these revisions satisfactorily address the Reviewer's concern while preserving the scientific rigor of the manuscript.

Referee #2:

General comments

The manuscript reports on the efficacy of cycle ergometry as a spaceflight countermeasure. In addition, the study investigated whether the outcome of a daily cycle ergometry exercise would be improved, were the exercise conducted during artificial gravity (AG). AG was established with a short arm human centrifuge. This study was part of a larger study supported by the European Space Agency: Bedrest, Artificial gravity and Cycle Exercise, or BRACE for short.

Subjects were confined to 6° head down bed rest for 60 days, and were separated into 3 groups: a control group, a group that conducted the cycle ergometry exercise in the horizontal position, and a group that conducted the cycle ergometry exercise in the horizontal position, while being exposed to a head-to-foot gravitational vector during centrifugation. The results indicate that both exercise countermeasures were not capable of mitigating the loss of muscle mass and strength but preserved metabolic stability.

The article is well written; the results clear and the conclusions are appropriate.

Impact on the area of research

Considering the new era of space travel, both explorative (missions to the Moon and Mars) and touristic (low Earth orbit) in nature, the science of human spaceflight must match the engineering developments. An optimal countermeasure has not yet been developed, that would prevent adaptation of physiological systems to weightlessness and/or reduced gravity. The development of such countermeasures is a slow and arduous process. This manuscript evaluates a new strategy, that of artificial gravity combined with cycle ergometry. As a consequence, the report is extremely important in the field of exercise countermeasures, but not only for space travel, but also for populations on Earth that exhibit muscle loss (i.e., age related muscle atrophy).

The research is highly original. To my knowledge, no long/term studies have been conducted on healthy young inactive individuals to assess the benefit of artificial gravity during cycle ergometry.

AR: We thank Referee #2 for their careful and constructive evaluation of our manuscript. We greatly appreciate their positive assessment of the study's originality, clarity of presentation, and relevance to the field of exercise countermeasures for spaceflight. We are also grateful for their recognition of the broader implications of this work, not only for human space exploration but also for terrestrial populations affected by muscle loss. The detailed comments provided have been very helpful in improving the clarity, methodological transparency, and physiological interpretation of the manuscript. We have addressed all points raised below and believe that the revised version has been substantially strengthened as a result.

Major comments

Q: 1. The determination of the energy intake of the individual participants and the three groups is not clear. The section describing the determination of energy intake is confusing.

AR: We thank the reviewer for their careful reading of the nutritional prescription section. We have substantially revised this part of the manuscript to clarify (i) how individual energy requirements were calculated, (ii) how prescriptions evolved across study phases, and (iii) how macronutrient intake was standardized between groups. We have also added explicit information regarding the food composition databases used and clarified the differences in caloric intake between groups. A summary table of mean energy and macronutrient intake by period and group is now referenced in the Results.

Briefly, energy intake was individualized based on measured RMR at BDC-14 and expressed as a Physical Activity Level (PAL) multiplier. Prescriptions were phase-specific (BDC/Recovery PAL = 1.60; HDT1–HDT10 PAL = 1.30; HDT11–HDT49 revised PAL = 1.42; HDT50–HDT60 PAL = 1.49). Exercise-related caloric compensation was based on the estimated mechanical workload and was applied identically in the EX and EX-AG groups.

Nutritional composition was calculated using validated French CIQUAL food composition databases supplemented by manufacturer information. Macronutrient distribution was standardized across groups throughout the study. Protein intake was prescribed at 1.0–1.2 $g \cdot kg^{-1} \cdot day^{-1}$ (1.3 $g \cdot kg^{-1}$ during the flexible period), fat contributed ~35% of total energy, and carbohydrate provided the remaining energy.

Actual energy intake closely matched prescription targets across study phases, and macronutrient distribution remained comparable between groups (Table 4).

Q: 2. The prescription of the exercise is vague. This study evaluates a training programme comprised of cycle ergometry. Presumably participants received personalized training programmes. How was this determined?

AR: We thank the Reviewer for highlighting the need for greater clarity regarding the prescription and individualization of the exercise intervention. We agree that the description of the exercise protocol was insufficiently detailed in the original submission.

We have now substantially expanded the countermeasures section to clearly describe how exercise intensity was individually prescribed based on supine $\dot{V}O_2$peak, and how the artificial gravity protocol was tailored to each participant using orthostatic tolerance and presyncope thresholds.

These revisions clarify that the intervention consisted of a standardized exercise countermeasure with individualised intensity prescription, rather than a progressive training programme.

Q: 3. Participants in the artificial gravity groups were most likely experiencing some effects of the centrifugation (i.e., motion sickness). Did this affect their training programme compared to the EX-group? Was the training the same for both groups? Any reports of muscle soreness that may have impacted on the ability of the participants to conduct the daily exercise? Did the motion sickness affect their appetite?

AR: We thank the Reviewer for raising this important point regarding potential side effects of centrifugation and their impact on training adherence and performance. Motion sickness symptoms were systematically monitored during the study using the Rating Scale Questionnaire (RSQ), which was administered immediately after each countermeasure session (or at the equivalent time point in the control group), in line with the BRACE neurovestibular monitoring protocol.

A review of the RSQ database across both BRACE campaigns indicated that symptoms of motion sickness during artificial gravity exposure were minimal and transient, with no evidence of clinically relevant discomfort that would have interfered with exercise execution. Importantly, there were no systematic differences between the EX and EX-AG groups in the ability to complete the prescribed training sessions.

This is further supported by the countermeasure deviation logs, which show that nearly all exercise sessions were performed as planned in both intervention groups, indicating excellent adherence and no meaningful disruption of the training programme due to centrifugation-related symptoms.

Muscle soreness was not reported as a limiting factor for training compliance, and there were no indications that artificial gravity exposure adversely affected appetite or energy intake, which were closely monitored and regulated throughout the intervention.

Overall, these observations suggest that artificial gravity was well tolerated and did not compromise training quality, volume, or adherence compared with exercise alone.

Minor comments

Q: Line 116: This is essentially a study evaluating an exercise training programme. As such, it would benefit the reader to have some more detailed information regarding the exercise protocol. For example, what is meant by "standardised physical activity"? If the subjects were confined, how could they be very active?

AR: We thank the Reviewer for this important comment and agree that additional detail was required to clarify the physical activity performed during the baseline data collection (BDC) period. The term "standardised physical activity" referred to a structured and supervised activity programme implemented during BDC, prior to the head-down tilt bed rest phase, and not to the period of immobilisation.

During BDC, participants were confined to the research facility but were not immobilised. Physical activity was prescribed to maintain cardiovascular fitness and prevent pre-bed-rest deconditioning. The activity programme followed a standardised framework, while the total volume of activity was individualised according to each participant's habitual activity-related energy expenditure, calculated from resting metabolic rate assessments.

Approximately 40% of each participant's habitual activity energy expenditure was achieved through structured exercise sessions performed within the facility. These sessions included:

- Treadmill exercise, consisting of a 5-min warm-up walking at 5 $km \cdot h^{-1}$, followed by 5 min walking at 6 $km \cdot h^{-1}$, and then running at 9 $km \cdot h^{-1}$, with the duration of the running phase individually adjusted according to each participant's energy expenditure and body mass.
- Cycle ergometry, with a total duration of 40 min, including 5 min at 50 W, 5 min at 90 W, and 30 min at a resistance individually adjusted based on habitual energy expenditure and body mass.

The remaining ~60% of individual habitual activity energy expenditure was achieved through walking within the facility. Daily step counts were monitored using a wrist-worn activity monitor (Polar Loop), ensuring that prescribed activity levels were reached. All exercise sessions and physical activity were supervised by MEDES staff. We have now expanded the Methods section to fully describe the BDC physical activity protocol and to clarify the meaning of “standardised physical activity” in this context.

This new information has been added in “participants and facilities” section.

Q: Line 117: By "countermeasure" you mean exercise training. Although perhaps redundant for the space life scientists, it might be helpful to define this for others.

AR: We thank the Reviewer for this helpful suggestion. We have now defined the term “countermeasure” at its first occurrence in the introduction section to clarify that it refers to structured interventions implemented to mitigate physiological deconditioning during prolonged bed rest, thereby improving accessibility for readers outside the space life sciences field.

Q: Line 129: What was the "standardised rehabilitation programme"? The term "standardised" is used throughout the text. Does this imply "personalised"? Was the physical activity (see above) before the bedrest the same for all subjects? Was the exercise training intensity the same for all subjects?

AR: We thank the Reviewer for this important point regarding the use of the term “standardised”. Throughout the manuscript, “standardised” refers to the use of a common, pre-defined framework applied consistently across participants, rather than to identical workloads or fully individualised prescriptions.

Before bed rest, physical activity during the baseline data collection phase followed a standardised and supervised programme, while the total activity volume was individualised based on each participant’s habitual activity-related energy expenditure, as now clarified in the Methods. Similarly, during the post-bed rest recovery phase, all participants followed a standardised rehabilitation programme delivered according to a common protocol, with progression adapted to individual tolerance. This rehabilitation period was implemented for ethical and clinical reasons and was not designed as an experimental intervention nor analysed as a study outcome. We have revised the Methods (participants and facilities) section to clarify this distinction.

Q: Line 138 - 141: Presumably in such a confinement study incorporating an exercise training protocol, the energy balance is of utmost importance. What food data bases were used to determine the energy intake? The micronutrients were controlled, as explained later in the text, what about macronutrients? What was the difference in the caloric intake between the groups? was there a large individual difference between subjects?

AR: see below.

Q: Line 151: You state that "early monitoring of body mass and composition" suggested that the energy requirements were underestimated. In the previous paragraph it is stated that the energy intakes were modified due to the requirements of a "scientific team"? Firstly, it might be helpful to explain that other teams were involved in the study. Secondly, this section requires much more detail. Body mass and composition is a function of the daily activity and energy intake (quality and quantity). From the description provided, it would appear that only the exercise was controlled. There is no discussion provided on how this variability in the energy intake affected body mass and composition.

AR: see below

Q: Perhaps a timeline would help in understanding this. Participants were provided with an energy intake of 130% of resting metabolic rate during HDT 1 to HDT 11. They were then on a new schedule from HDT 11 to HDT 51, whereby the energy intake was between 90 and 110% of estimated requirements. Whan was the energy intake increased to 142%?

AR: see below

Q: Line 156: Was it 1 or 1.2? Did it differ for the participants? Groups?

AR: see below

Q: Line 159: What about the macronutrient recommendations?

AR: We thank the Reviewer for their careful and thorough reading of the nutritional prescription section and for highlighting the need for greater clarity regarding energy balance and dietary control. In response to all these five comments, we have substantially revised and expanded the nutritional prescription subsection of the Methods.

The revised text now provides a detailed and phase-specific description of energy prescription across the different stages of the bed rest protocol, including the rationale for adjustments based on early monitoring of body mass and composition, and clarifies the involvement of multiple scientific teams in the overall study design. It also explicitly details the control and standardisation of macronutrient intake, in addition to micronutrients, and explains how individual variability in energy intake was managed within predefined limits.

Differences in total caloric intake between groups, driven by exercise-related energy compensation, are now clearly described, while macronutrient distribution remained comparable across groups by design. To facilitate interpretation, a summary table of mean energy and macronutrient intake by period and group is now referenced in the Results section.

We believe that these revisions address all the Reviewer's questions regarding energy prescription, dietary composition, inter-individual variability, and their potential impact on body mass and composition.

Q: Line 171: You use the term orthostasis during centrifugation. Normally, orthostasis refers to the physiological responses to standing upright, whereas g-tolerance is the response to elevated head to foot gravitational vector. Is OT the correct term here? Orthostatic tolerance is normally assessed by tilting the participant to 80° upright and applying a reduced pressure around the lower body (lower body reduced/negative pressure). I am not sure how this measurement is critical for your results?

AR: We use orthostatic tolerance only to refer to the countermeasure protocol design. This is the term originally used in the experiments performed to determine the AG load for BRACE (Ap et al., 2025; reference 14). Therefore, we argue it is important to use the same terminology to avoid potential misunderstandings. We hope the reviewer shares this approach.

Q: Line 181: Was the exercise conducted as a percentage of the upright or supine VO2max? Please define. With time the daily training most likely modified the relative workload compared to the absolute workload. In other words, 40% of VO2max at the start of the bed rest may have change by the end of the bed rest. In the event that there was a positive effect, then this may be reduced (i.e., to 30% VO2max), or increased as a result of a negative effect. Was the VO2max measured at the end of the bed rest? Was the exercise intensity modified during the bed rest, as it would be during a training programme?

AR: We thank the Reviewer for this important comment. Exercise intensity was prescribed based on VO_2peak measured during a supine incremental cycling test performed prior to the head-down tilt bed rest period. Exercise workloads were maintained constant in absolute terms throughout the intervention, and VO_2peak was not reassessed for the purpose of adjusting exercise intensity during bed rest. We acknowledge that this represents a limitation of the countermeasure design, as the relative exercise intensity may have changed over time due to bed rest–induced physiological adaptations.

This design choice was made to ensure standardisation of the countermeasure across participants and to minimise interference with other scientific protocols implemented during the BRACE study. Importantly, this limitation should be considered when interpreting the dissociation observed between the preservation of resting metabolic rate and the lack of protection of neuromuscular performance. We have now explicitly acknowledged this limitation in the Discussion section.

Q: Line 236: The Quark device has an alcohol burner for calibrating the RMR determined with the canopy. Was the gas calibration conducted in addition to the alcohol calibration? Could you be more specific as to how the gas calibration was conducted? Was this conducted with the canopy positioned on a surface (i.e., pillow)? The Quark system draws the gas from the canopy at a predetermined rate. By introducing an additional flow of gas into the canopy are you not affecting the measurement of flow?

AR: We thank the reviewer for this important question. In the present study, we did not perform alcohol burn calibration in addition to the gas-based simulation protocol. Instead, a dedicated simulation system was used to reproduce controlled and physiologically relevant gas exchange conditions. Importantly, reference gas was not infused directly into the canopy. Gas mixtures were first generated within an external mixing chamber, where compressed reference gas (CO_2/N_2) was blended with ambient air under controlled conditions using a precision mass flow controller. The mixed gas was then aspirated by the Quark system at predefined ventilation flows (25 and 45 $L \cdot min^{-1}$), thereby reproducing subject-like breathing conditions without introducing external flow into the canopy itself. This configuration ensured that total flow through the system remained entirely controlled by the device pump and was not artificially altered by gas infusion. Eight simulation points were tested (four at each ventilation level), spanning a range of FeO_2 and $FeCO_2$ values representative of physiological resting conditions. Theoretical VO_2 and VCO_2 values were derived from known input flows and gas fractions, allowing regression-based correction factors to be calculated. The simulation protocol allowed reproduction of physiologically plausible respiratory exchange ratios (~0.85) without combustion and provided stable reference conditions for drift correction across sessions and devices.

Q: Line 258: Was familiarization conducted before the actual session (on another day) or just prior to the measurement?

AR: Familiarization was conducted prior to the measurements.

Q: Line 365: Since blood pressure was measured at rest, the measurement is not a "response" to a stimulus. Was the blood pressure response to the exercise affected? It would be helpful, if you reported the heart rate and blood pressure during the exercise over the 60-d period.

AR: We thank the Reviewer for this helpful clarification. We agree that, as blood pressure and heart rate were measured under standardized resting conditions, these measurements should not be described as a "response" to an acute stimulus.

In the present study, resting heart rate and blood pressure were assessed each morning upon awakening, in the fasted supine state, with the aim of characterizing basal cardiovascular adaptations to prolonged bed rest and the countermeasure interventions. We have revised the text to clarify this point and to avoid the use of terminology that may imply an acute exercise response.

We believe that the revised wording more accurately reflects the nature of the measurements and their interpretation.

Q: Line 546: How would the varying energy intake affect RMR and substrate utilisation?

AR: We thank the Reviewer for raising this important point. Variations in energy intake can indeed influence RMR and substrate utilisation, particularly under conditions of prolonged inactivity. For this reason, dietary intake was tightly controlled throughout the study, with all

meals weighed, standardised menus applied, and energy prescriptions derived from individually measured RMR. Complete consumption was usually required, with dinner adjustments implemented when necessary. During the head-down tilt period, a phase of controlled flexibility in energy intake (90–110% of estimated requirements) were implemented as part of the overarching BRACE protocol. But, if energy intake fell below 90% of the prescribed target for two consecutive days, participants were required to consume 100% of their prescribed energy requirement on the third day to maintain compliance with the energy needs. Importantly, adjustments of energy prescription targets were applied across groups and were introduced to better match actual energy requirements as inferred from early changes in body mass and composition. At no point did any group exhibit sustained positive energy balance.

From a physiological perspective, a reduction in energy intake would be expected to lower RMR and promote a shift toward greater fat oxidation, whereas energy surplus would tend to increase carbohydrate utilisation and RQ. However, the observed metabolic responses did not follow this pattern. Despite comparable dietary prescriptions, the control and EX groups exhibited marked declines in RMR accompanied by increases in RQ and carbohydrate utilisation, whereas the EX-AG group maintained RMR and preserved fasting fat oxidation. Crucially, between-group differences in RMR and substrate utilisation persisted after adjustment for concurrent fat-free mass, indicating that changes in tissue quantity alone, or differences in dietary intake per se, cannot fully account for the observed metabolic patterns. Multivariate analyses further identified changes in respiratory quotient as the dominant contributor to ΔRMR, supporting the interpretation that preservation of metabolic flexibility and oxidative metabolism underlies the maintenance of RMR in the EX-AG group.

Taken together, while minor variability in energy intake may have contributed to inter-individual variability, the consistency of dietary control across groups and the persistence of metabolic differences after FFM adjustment suggest that the preservation of RMR and lipid utilisation in EX-AG primarily reflects physiological adaptations to combined exercise and artificial gravity rather than dietary effects.

Q: Line 557: The authors state that "artificial gravity likely increased cardiovascular.... demands". Were the cardiovascular responses not monitored during the exercise?

AR: We thank the Reviewer for this important point. Cardiovascular responses (e.g., heart rate and blood pressure) were not systematically monitored during exercise sessions across the intervention period in a manner suitable for quantitative or longitudinal analysis.

The statement regarding increased cardiovascular demands under artificial gravity was therefore intended as a physiological inference based on established responses to head-to-foot gravitational

loading, rather than as a direct observation. We have revised the Discussion to explicitly clarify this point, rephrasing the statement to reflect its inferential nature and acknowledging the absence of direct cardiovascular measurements during exercise as a limitation.

Q: Table 1: The title of the Table suggests that the DXA values are compared by group at R+3 and BDC-1. The BDC column should then read BDC-1. The Table has two additional columns HDT60 and ΔHDT60. Should the title of the Table be modified to reflect this? Where was the moving average applied? Would it not be better to indicate that the Table represents values relative to BDC-1, rather than a "comparison"?

AR: see below.

Q: Table 2: Indirect calorimetry values at HDT60 relative to baseline?

AR: see below.

Q: Table 3: I am no sure I understand this Table. The title suggests that it presents indirect calorimetry values adjusted for FFM at HDT60 relative to baseline. There are no indirect calorimetry values in this Table, only DXA values? Also, there are no values for HDT60, but for HDT10, HDT44, HDT56 the BDC column should probably be defined as BDC-1). If these values are relative to BDC, why have a BDC column?

AR: We thank the Reviewer for their careful examination of Tables 1–3 and for highlighting the lack of clarity in their presentation. We agree that the issues raised stemmed from an inconsistency between table numbering, titles, and content in the submitted version of the manuscript.

Specifically, during submission, the order of Tables 1–3 did not match the list of table titles and the corresponding references in the text, which resulted in confusion regarding the reported variables, time points, and reference conditions. We apologize for this mismatch, which has now been fully corrected.

In the revised manuscript, table numbering, titles, and content have been realigned to ensure internal consistency and clarity. Baseline columns have been consistently defined as BDC-1 for calorimetric variables and BDC-2 for DXA-derived body-composition measures, in accordance with the study design. Table titles have been modified to explicitly indicate when values represent absolute measurements at specific time points or changes relative to baseline, rather than between-group "comparisons."

Tables presenting changes over time now explicitly report values relative to baseline, and redundant baseline columns have been removed where appropriate to avoid ambiguity. We have also clarified in the table titles and footnotes how moving averages and adjusted values were derived.

We believe that these revisions fully address the Reviewer's concerns and substantially improve the clarity and interpretability of the tables.

Dear Dr Fernandez-Gonzalo,

Re: JP-RP-2026-290788R1 "Artificial Gravity During Prolonged Bed Rest Preserves Resting Metabolic Rate but not Muscle Function: Evidence from the BRACE Study" by Saul Martin Rodriguez, Mirko Mandić, Claire Laurens, Marie-Pierre Bareille, Rebecca Billette de Villemeur, Rodrigo Fernandez-Gonzalo, and Chantal Simon

Thank you for submitting your manuscript to The Journal of Physiology. It has been assessed by a Reviewing Editor and by an expert referee and we are pleased to tell you that it is acceptable for publication following satisfactory revision.

The referee reports are copied at the end of this email.

Please address all the points raised and incorporate all requested revisions or explain in your Response to Referees why a change has not been made. We hope you will find the comments helpful and that you will be able to return your revised manuscript within 4 weeks. If you require longer than this, please contact journal staff: jp@physoc.org.

Your revised manuscript should be submitted online using the link in your Author Tasks Link Not Available. This link is accessible via your account as Corresponding Author; it is not available to your co-authors. If this presents a problem, please contact journal staff (jp@physoc.org). Image files from the previous version are retained on the system. Please ensure you replace or remove any files that are being revised.

If you do not wish to submit a revised version of your manuscript, you must inform our journal staff (jp@physoc.org) or reply to this email to request withdrawal. Please note that a manuscript must be formally withdrawn from the peer review process at one journal before it may be submitted to another journal.

TRANSPARENT PEER REVIEW POLICY: To improve the transparency of its peer review process The Journal of Physiology publishes online, as supporting information, the peer review history of all articles accepted for publication. Readers will have access to decision letters, including Editors' comments and referee reports, for each version of the manuscript, as well as any author responses to peer review comments. Referees can decide whether or not they wish to be named on the peer review history document.

ABSTRACT FIGURES: Authors are expected to use The Journal's premium BioRender account to create/redraw their Abstract Figures. Information on how to access this account is here: https://physoc.onlinelibrary.wiley.com/journal/14697793/biorender-access.

This will enable Authors to create and download high-resolution figures. If authors have used the free BioRender service, they can use the instructions provided in the link above to download a high-resolution version suitable for publication.

The link provided should only be used for the purposes of this submission. Authors will be charged for figures created on this account if they are not related to this manuscript submission.

LANGUAGE EDITING AND SUPPORT FOR PUBLICATION: If you would like help with English language editing, or other article preparation support, Wiley Editing Services offers expert help, including English Language Editing, as well as translation, manuscript formatting, and figure formatting at www.wileyauthors.com/eeo/preparation. You can also find resources for Preparing Your Article for general guidance about writing and preparing your manuscript at www.wileyauthors.com/eeo/prepresources.

RIGOUR AND REPRODUCIBILITY: We recommend authors consult The Journal's Rigour and Reproducibility requirements to ensure that their manuscript is suitable for publication: https://physoc.onlinelibrary.wiley.com/pb-assets/hub-assets/physoc/documents/TJP-Rigour-and-Reproducibility-Requirements.pdf

REVISION CHECKLIST:

Check that your Methods section conforms to journal policy: https://jp.msubmit.net/cgi-bin/main.plex?form_type=display_requirements#methods

Check that data presented conforms to the statistics policy: https://jp.msubmit.net/cgi-bin/main.plex?form_type=display_requirements#statistics

Upload a full Response to Referees file. To create your 'Response to Referees' copy all the reports, including any comments from the Senior and Reviewing Editors, into a Microsoft Word, or similar, file and respond to each point, using font or background colour to distinguish comments and responses and upload as the required file type.

Please upload two versions of your manuscript text: one with all relevant changes highlighted and one clean version with no changes tracked. The manuscript file should include all tables and figure legends, but each figure/graph should be uploaded as separate, high-resolution files. The journal is now integrated with Wiley's Image Checking service. For further details, see: https://www.wiley.com/en-us/network/publishing/research-publishing/trending-stories/upholding-image-integrity-wileys-

image-screening-service

You may also upload:

- 'Potential Cover Art' for consideration as the issue's cover image
- Appropriate Supporting Information (Video, audio or data set: see https://jp.msubmit.net/cgi-bin/main.plex?form_type=display_requirements#supp).

We look forward to receiving your revised submission.

If you have any queries, please reply to this email and we will be pleased to advise.

Yours sincerely,

Karyn Hamilton
Senior Editor
The Journal of Physiology

REQUIRED ITEMS FOR REVISION

- The reference list must be in alphabetical order, rather than numbered, to comply with our Journal format.

- Your manuscript must include a complete Additional Information section, including competing interests; funding; author contributions and acknowledgements.

- Please include an Abstract Figure file and an Abstract Figure legend. An appropriate figure legend, which should not exceed 150 words in length, should be included in the main manuscript file. The Abstract Figure is a piece of artwork designed to give readers an immediate understanding of the research and should summarise the main conclusions. If possible, the image should be easily 'readable' from left to right or top to bottom. It should show the physiological relevance of the manuscript so readers can assess the importance and content of its findings. Abstract Figures should not merely recapitulate other figures in the manuscript. Please try to keep the diagram as simple as possible and without superfluous information that may distract from the main conclusion(s). Abstract Figures must be provided by authors no later than the revised manuscript stage and should be uploaded as a separate file during online submission labelled as File Type 'Abstract Figure'. Please also ensure that you include the figure legend in the main article file. All Abstract Figures should be created using BioRender. Authors should use The Journal's premium BioRender account to export high-resolution images. Details on how to use and access the premium account are included as part of this email.

EDITOR COMMENTS

Reviewing Editor:

Thank you for considering and responding to the reviewers previous comments. There are a few minor points for you to consider from the reviewer based on the updated manuscript. Could you also in your figure legends indicate the number of participants included in the figures.

Senior Editor:

Thank you for submitting your revised manuscript for continued consideration by The Journal of Physiology. The Referees were complimentary about the improvements resulting from the revisions. Only a few minor suggestions remain. At this point, we would like to provisionally accept the manuscript pending satisfactory revisions to address these few remaining points. We look forward to seeing your revisions soon. Thank you again!

REFEREE COMMENTS

Referee #2:

I would like to thank the authors for a comprehensive revision of the manuscript.

I would have some last comments, which I apologise I did not make in the first review of the manuscript. The issue is with the measurement of the vital signs and the Table displaying the results.

Line 297 to 299: You list the measurements conducted, but do omit to mention the instruments (equipment) used. You might also consider labelling these measurements (i.e., Tty, HR, SAP, DAP MAP, etc.) The reason for his suggestion is, because in Table 1 you use labels, which have not been defined.

In table 1, I would suggest the following:

• Instead of Body temperature, use Tty, or Tympanic temperature or Core temperature. The proper unit is {degree sign}C.

• The unit for heart rate (HR) is min-1.

• Presumably PAS, PAD PAM are abbreviations of the French definition. In Engligh these should read SAP (systolic arterial pressure), DAP (diastolic arterial pressure) and MAP (mean arterial pressure.

END OF COMMENTS

EDITOR COMMENTS

Reviewing Editor:

Thank you for considering and responding to the reviewers previous comments. There are a few minor points for you to consider from the reviewer based on the updated manuscript. Could you also in your figure legends indicate the number of participants included in the figures.

Senior Editor:

Thank you for submitting your revised manuscript for continued consideration by The Journal of Physiology. The Referees were complimentary about the improvements resulting from the revisions. Only a few minor suggestions remain. At this point, we would like to provisionally accept the manuscript pending satisfactory revisions to address these few remaining points. We look forward to seeing your revisions soon. Thank you again!

Authors' response (AR): We thank the Reviewing and Senior Editors for their assessment of our manuscript and for the constructive guidance provided. We are pleased to have received provisional acceptance.

In the revised version, we have addressed the minor comments raised by Reviewer #2 and implemented the following changes: participant numbers have been added to all figure legends; a thorough language check has been performed, correcting typos and grammatical errors; the abstract has been revised to comply with the word limit; references have been reformatted according to Journal guidelines; and a complete Additional Information section has been included, covering competing interests, funding, author contributions, and acknowledgements.

We believe that with these changes the revised manuscript meets the Journal's requirements for publication.

REFEREE COMMENTS

Referee #2:

Reviewer's comment (RC): I would like to thank the authors for a comprehensive revision of the manuscript.

I would have some last comments, which I apologise I did not make in the first review of the manuscript. The issue is with the measurement of the vital signs and the Table displaying the results.

AR: We thank the reviewer for her/his past and present feedback, which has clearly improved the quality and clarity of the paper.

RC: Line 297 to 299: You list the measurements conducted, but do omit to mention the instruments (equipment) used. You might also consider labelling these measurements (i.e., Tty, HR, SAP, DAP MAP, etc.) The reason for his suggestion is, because in Table 1 you use labels, which have not been defined.

AR: Instruments used have been added, with the corresponding manufacturer information. The labelling for these measurements has been modified according to the reviewer's comment, including Table 1.

RC: In table 1, I would suggest the following:

• Instead of Body temperature, use Tty, or Tympanic temperature or Core temperature. The proper unit is {degree sign}C.

• The unit for heart rate (HR) is min-1.

• Presumably PAS, PAD PAM are abbreviations of the French definition. In Engligh these should read SAP (systolic arterial pressure), DAP (diastolic arterial pressure) and MAP (mean arterial pressure.

AR: The changes in Table 1 suggested by the reviewer have been implemented.

Dear Dr Fernandez-Gonzalo,

Re: JP-RP-2026-290788R2 "**Artificial Gravity During Prolonged Bed Rest Preserves Resting Metabolic Rate but not Muscle Function: Evidence from the BRACE Study**" by Saul Martin Rodriguez, Mirko Mandić, Claire Laurens, Marie-Pierre Bareille, Rebecca Billette de Villemeur, Rodrigo Fernandez-Gonzalo, and Chantal Simon

We are pleased to tell you that your paper has been accepted for publication in The Journal of Physiology.

The last Word (or similar) version of the manuscript provided will be used by the Production Editor to prepare your proof. When this is ready you will receive an email containing a link to Wiley's Online Proofing System. The proof should be thoroughly checked and corrected as promptly as possible.

Authors should note that it is too late at this point to offer corrections prior to proofreading. Major corrections at proof stage, such as changes to figures, will be referred to the Editors for approval before they can be incorporated. Only minor changes, such as to style and consistency, should be made at proof stage. Changes that need to be made after proof stage will usually require a formal correction notice.

All queries at proof stage should be sent to: TJP@wiley.com.

Following copyediting and typesetting the accepted version will be published online.

Yours sincerely,

Karyn Hamilton
Senior Editor
The Journal of Physiology

IMPORTANT POINTS TO NOTE FOLLOWING ACCEPTANCE OF YOUR PAPER:

• IMPORTANT NOTICE ABOUT OPEN ACCESS: To assist authors whose funding agencies mandate immediate public access to published research findings, The Journal of Physiology allows authors to pay an Open Access (OA) fee to have their papers made freely available immediately on publication.

The Corresponding Author will receive an email from Wiley with details on how to register or log in to Wiley Authors where you will be able to place an order.

• You can check if your funder or institution has a Wiley Open Access Account here:
https://authors.wiley.com/author-resources/Journal-Authors/open-access/author-compliance-tool.html

• TRANSPARENT PEER REVIEW POLICY: To improve the transparency of its peer review process, The Journal of Physiology publishes online as supporting information the peer review history of all articles accepted for publication. Readers will have access to decision letters, including Editors' comments and referee reports, for each version of the manuscript as well as any author responses to peer review comments. Referees can decide whether or not they wish to be named on the peer review history document.

• You can help your research get the attention it deserves! Check out Wiley's free Promotion Guide for best-practice recommendations for promoting your work at: www.wileyauthors.com/eeo/guide. You can learn more about Wiley Editing Services which offers professional video, design, and writing services to create shareable video abstracts, infographics, conference posters, lay summaries, and research news stories for your research at: www.wileyauthors.com/eeo/promotion.

• If you would like to receive our 'Research Roundup', a monthly newsletter highlighting the cutting-edge research published in The Physiological Society's family of journals (The Journal of Physiology, Experimental Physiology, Physiological Reports, The Journal of Nutritional Physiology and The Journal of Precision Medicine: Health and Disease), please click this link, fill in your name and email address and select 'Research Roundup':
https://www.physoc.org/journals-and-media/membernews

EDITOR COMMENTS

Reviewing Editor:

Thank you for considering/addressing the comments from the reviewers and editors. Overall the manuscript reads well and presents an interesting study.

Senior Editor:

Thank you for submitting your revised manuscript. We are pleased to accept it for publication in The Journal of Physiology. Thank you for your interest in The Journal and Congratulations!
